# Actin-nucleation promoting factor N-WASP influences alpha-synuclein condensates and pathology

**DOI:** 10.1038/s41419-024-06686-7

**Published:** 2024-04-30

**Authors:** Joshua Jackson, Christian Hoffmann, Enzo Scifo, Han Wang, Lena Wischhof, Antonia Piazzesi, Mrityunjoy Mondal, Hanna Shields, Xuesi Zhou, Magali Mondin, Eanna B. Ryan, Hermann Döring, Jochen H. M. Prehn, Klemens Rottner, Gregory Giannone, Pierluigi Nicotera, Dan Ehninger, Dragomir Milovanovic, Daniele Bano

**Affiliations:** 1 German Center for Neurodegenerative Diseases (DZNE), Bonn, Germany; 2 German Center for Neurodegenerative Diseases (DZNE), Berlin, Germany; 3 Einstein Center for Neuroscience, Charité-Universitätsmedizin Berlin, Corporate Member of Freie Universität Berlin, Humboldt-Universität Berlin, and Berlin Institute of Health, Berlin, Germany; 4 University Bordeaux, CNRS, Interdisciplinary Institute for Neuroscience, IINS, UMR 5297, Bordeaux, France; 5 University Bordeaux, CNRS, INSERM, BIC, UAR 3420, F-33000 Bordeaux, France; 6 RCSI Centre for Systems Medicine and Department of Physiology and Medical Physics, RCSI University of Medicine and Health Sciences; SFI FutureNeuro Research Centre, Dublin 2, Ireland; 7 Division of Molecular Cell Biology, Zoological Institute, Technische Universität Braunschweig; Department of Cell Biology, Helmholtz Centre for Infection Research, Braunschweig, Germany

**Keywords:** Parkinson's disease, Mechanisms of disease, Protein-protein interaction networks

## Abstract

Abnormal intraneuronal accumulation of soluble and insoluble α-synuclein (α-Syn) is one of the main pathological hallmarks of synucleinopathies, such as Parkinson’s disease (PD). It has been well documented that the reversible liquid-liquid phase separation of α-Syn can modulate synaptic vesicle condensates at the presynaptic terminals. However, α-Syn can also form liquid-like droplets that may convert into amyloid-enriched hydrogels or fibrillar polymorphs under stressful conditions. To advance our understanding on the mechanisms underlying α-Syn phase transition, we employed a series of unbiased proteomic analyses and found that actin and actin regulators are part of the α-Syn interactome. We focused on Neural Wiskott-Aldrich syndrome protein (N-WASP) because of its association with a rare early-onset familial form of PD. In cultured cells, we demonstrate that N-WASP undergoes phase separation and can be recruited to synapsin 1 liquid-like droplets, whereas it is excluded from α-Syn/synapsin 1 condensates. Consistently, we provide evidence that *wsp-1/WASL loss of function* alters the number and dynamics of α-Syn inclusions in the nematode *Caenorhabditis elegans*. Together, our findings indicate that N-WASP expression may create permissive conditions that promote α-Syn condensates and their potentially deleterious conversion into toxic species.

## Introduction

Synucleinopathies are a clinically heterogeneous group of neurodegenerative diseases in which misfolded and/or aggregated α-Syn abnormally accumulates in cytoplasmic deposits generally known as Lewy bodies (LB) [1, 2]. Post-mortem assessment of patients with idiopathic PD or dementia with Lewy bodies (DLB) reveals α-Syn-positive deposits in well-defined regions, such as the *substantia nigra* (SN) *pars compacta*. The progressive accumulation of α-Syn-containing LB is associated with the degeneration of vulnerable neurons and the consequent onset of motor disabilities [3]. In addition to the presence of LB pathology, tissues from PD patients often show mitochondrial defects and signs of oxidative stress that are assumed to be part of the detrimental cascade leading to dopaminergic neuronal death [4]. While familial forms of PD have a clear etiology, idiopathic PD is considered a complex multifactorial age-related disorder in which aberrant α-Syn accumulation is one of the main hallmarks and causes of neurodegeneration [5]. Given the relevance in human pathophysiology, the molecular mechanisms underlying α-Syn pathology in PD and other synucleinopathies remain a topic of intense investigation, as they represent targets for therapeutic interventions.

Human α-Syn is an intrinsically disordered cytosolic protein consisting of 140 amino acids. Normally, α-Syn is highly expressed in neural cells and localizes at the presynaptic terminals, where it is highly abundant and modulates synaptic vesicle (SV) trafficking by promoting membrane curvature and chaperoning the formation of SNARE complexes [6, 7]. Recent evidence suggests that high concentrations of α-Syn promote its liquid-liquid phase separation (LLPS), with liquid-like droplets that could convert into amyloid-enriched hydrogel under certain conditions [8, 9]. Additionally, α-Syn condensates may act as reservoirs of highly concentrated α-Syn monomers that can contribute to the growth of fibrillar polymorphs, as it occurs in cells that are exposed to exogenous preformed fibrils [10]. It has been proposed that LLPS is a thermodynamic process in which macromolecules assemble into distinct subcellular compartments lacking a membrane or a scaffold [11]. In functionally healthy neurons, clusters of SVs at the synapses are shown to assemble by LLPS through their interaction with synapsins, a highly abundant family of synaptic phosphoproteins [12–14]. Synapsin 1 is an SV-associated protein that interacts with α-Syn when upregulated or overexpressed [15, 16]. Moreover, synapsin 1 establishes a functional coupling with α-Syn on the SV surface [17], thereby regulating vesicle recycling and abundance at the presynaptic terminals [18]. Although α-Syn partially retains its high mobility within the crowded environment at the nerve compartments, it is actively recruited and reversibly sequestered by SV/synapsin 1 condensates [19]. Interestingly, high molar ratios of α-Syn alter the assembly kinetics of SV/synapsin 1 condensates [19, 20], further indicating that expression changes of α-Syn may impact the biophysical properties of other synaptic molecules.

Clinical evidence indicates that duplication and triplication of *SNCA* gene can cause familial forms of PD [1]. Consistent with its toxic effect when aberrantly accumulated, it is known that α-Syn upregulation can occur upon stress or synaptic damage [21, 22], which may promote α-Syn mislocalization, interactions with non-conventional molecular partners and association with lipid bilayer membranes. Over time, these binding molecules can exert an influence on α-Syn’s conformational state, along with its recruitment and incorporation into functional liquid condensates or amyloid-like hydrogels [1, 6, 19, 23]. At least in experimental models, intracerebral inoculation of α-Syn toxic species or aggregates can rapidly lead to progressive neurodegeneration and parkinsonism [24, 25], further emphasizing the strong mechanistic link between aberrant α-Syn homeostasis and PD. Despite these functionally relevant consequences, the mechanisms that abolish the native localization of α-Syn from the synaptic boutons and drive its aberrant condensation in neuronal cell bodies remain unclear.

Given the contribution of α-Syn to PD and other neurodegenerative diseases, we hypothesized that a better understanding of the α-Syn interactome may help to reveal molecular processes linked to α-Syn LLPS and possibly to α-Syn toxicity. Thus, we set out a series of proteomic analyses and an unbiased in vitro protein-protein profiling of α-Syn interactors. We found that many α-Syn interaction partners are actin and actin-binding proteins, including members of the Wiskott-Aldrich syndrome (WAS) protein family, such as WASF1 and WASF3. We focused on the Neural Wiskott-Aldrich syndrome protein (N-WASP), an actin nucleation factor that binds and activates the Arp2/3 complex [26]. It is known that N-WASP can undergo liquid-like density phase transition [27, 28], which enhances its dwell time on the membrane and its positive influences on Arp2/3-dependent actin assembly [29]. Since compound heterozygous *WASL* mutations segregate in family members with early-onset PD [30], we sought to explore the biological consequence of N-WASP deficiency. We found that N-WASP expression can modify the size and number of α-Syn- and synapsin 1-containing liquid condensates. Furthermore, *wsp-1/WASL loss of function (lof)* lowers the tolerance of *Caenorhabditis elegans* to α-Syn overexpression. Together, these data describe the contribution of N-WASP to α-Syn homeostasis and LLPS, which may help to explain in part the early onset of PD in individuals expressing pathogenic N-WASP variants.

## Results

### Actin and actin-binding proteins are part of the α-Syn interactome

**Fig. 1:**
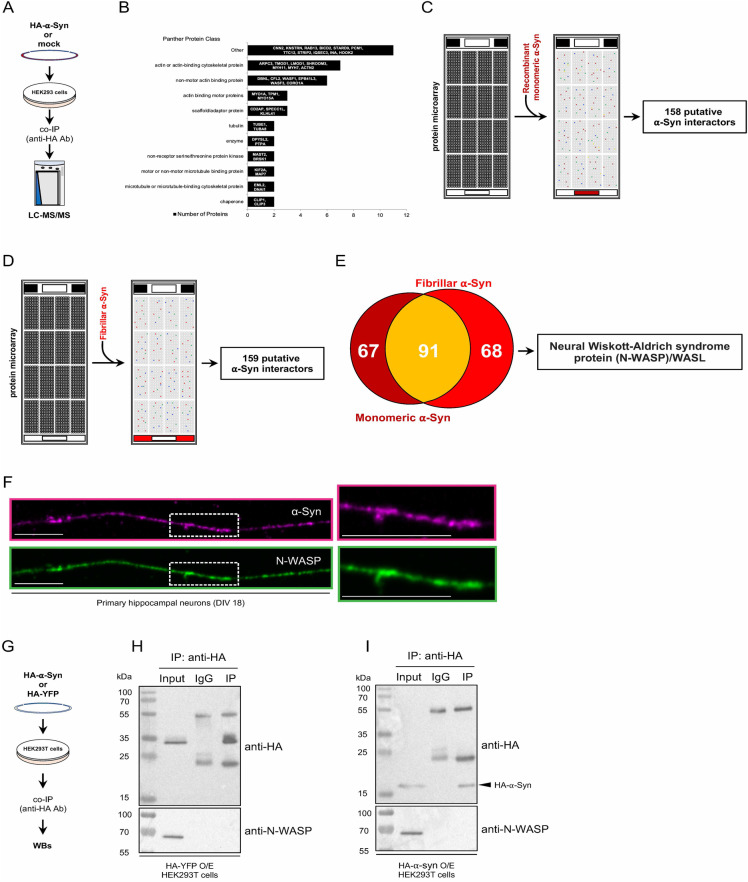
Proteomic analyses reveal novel α-Syn interactors. **A** Schematic representation of the co-immunoprecipitation experiment. Co-immunoprecipitated proteins were obtained using an antibody against HA, which was incubated with lysates from control and transiently transfected HEK293/Expi293 cells overexpressing HA-tagged α-Syn. Detection of α-Syn-interacting proteins was performed by LC–MS/MS. **B** Panther protein class of immunoprecipitated cytoskeleton-associated proteins. Schematic representation of the experimental workflow. Protein microchips were incubated with recombinant **C** monomeric or **D** fibrillar α-Syn and, after incubation with a primary antibody against α-Syn, developed using a fluorescent-labeled secondary antibody. Out of ~9000 polypeptides, **C** 158 and **D** 159 putative α-Syn-interacting polypeptides were retrieved. **E** Venn diagram of common and unique interactors of monomeric and/or fibrillar α-Syn. N-WASP was detected in both assays. **F** Representative image of a primary hippocampal neuron stained with antibodies against α-Syn (magenta) and N-WASP (green). Scale bars = 10 µm. **G** Schematic representation of co-IP strategy using HEK293T cells. Co-IPs were performed using homogenates of **H** HA-YFP O/E and **I** HA-α-Syn O/E HEK293T cells, which were incubated with an anti-HA antibody in the presence of Sepharose beads. Immunoblots were developed using anti-N-WASP and anti-HA antibodies.

To identify novel modulators of α-Syn LLPS in an unbiased manner, we initially performed co-immunoprecipitation experiments using HEK293 cells transiently overexpressing HA-tagged α-Syn. Upon incubation of cell homogenates with a validated HA antibody, we co-immunoprecipitated proteins that were then analyzed by liquid chromatography/mass spectrometry (LC–MS/MS) (Fig. 1A). We detected 347 proteins that were exclusively found in the HA-tagged α-Syn condition (Supplementary Table ST1). Of the detected proteins, 42 were actin, cytoskeletal proteins, and actin-binding factors (Supplementary Table ST1). Identified actin or actin-binding cytoskeletal proteins included ARPC3, TMOD1, LMOD1, SHROOM3, MYH11, MYH7 and ACTN2. Amongst the non-motor actin-binding proteins, we detected DBNL, CFL2, WASF1, EPB41L3, WASF3, and CORO1A (Fig. 1B and Supplementary Table ST1). GO BP analysis by ClueGO indicated that the 5 most significantly enriched terms included actin-myosin filament sliding (adj *p* value = 1.89 E-6) with 41.18% associated proteins (MYH2, MYH4, MYH7, MYH8, MYL1, MYL11, TPM1) detectable in our α-Syn co-IP dataset (Supplementary table ST2). In addition, 7.31% of the proteins (AGBL4, AP3M2, BICD2, CCDC186, CLIP3, HDAC6, HOOK2, KIF17, KIF1B, KIF5A, KIFAP3, MAK, MYO15A, MYO1A, PCM1, WASF1) linked to cytoskeleton-dependent intracellular transport (adj *p* value = 4.80 E-4) were also identified (Supplementary table ST2). Together, these data indicate that α-Syn has a certain binding propensity toward actin and other cytoskeletal factors.

To corroborate our findings using an additional method, we employed high-content protein microchips consisting of more than 9.000 human polypeptides. Following the same approach as described in one of our prior works [31], two separate microarrays were incubated with 5 or 50 ng of monomeric α-Syn. After development with primary and fluorescently labeled secondary antibodies, we identified 158 putative polypeptides that could physically interact with α-Syn in vitro (Fig. 1C and Supplementary Table ST3). Additionally, we carried out another protein microarray screen using fibrillar α-Syn and found 159 interacting polypeptides (Fig. 1D), of which 91 proteins were common hits with the monomeric α-Syn dataset (Fig. 1E and Supplementary Table ST3). Among these putative 91 α-Syn interactors, 5 proteins (GABARAPL2, NPM1, POLB, TWF1, WASL/N-WASP) were associated with the cytoskeleton and/or actin-binding factors, of which N-WASP was of particular interest because of its propensity to undergo a density phase transition and to bind the actin nucleator Arp2/3 complex [27, 29]. Given the causal relationship between pathogenic *WASL* mutations and inherited PD [30], we tested if N-WASP could physically bind α-Syn, since these two proteins highly co-localized in cultured cells (Fig. 1F). We set up a series of co-immunoprecipitations (co-IPs) using homogenates from HEK293T cells overexpressing HA-tagged YFP or HA-tagged α-Syn (Fig. 1G). After a mild exposure with the reversible crosslinker dithiobis (succinimidyl propionate) (DSP), we lysed the cells and carried out co-IPs using a validated primary antibody against HA. Subsequent SDS-PAGE and western blot analyses showed that N-WASP and α-Syn did not co-immunoprecipitate (Fig. 1H, I). This set of data suggests that α-Syn and N-WASP can physically bind in vitro, although their interaction is relatively weak and possibly transient in cultured cells.

### N-WASP forms condensates in cells

**Fig. 2:**
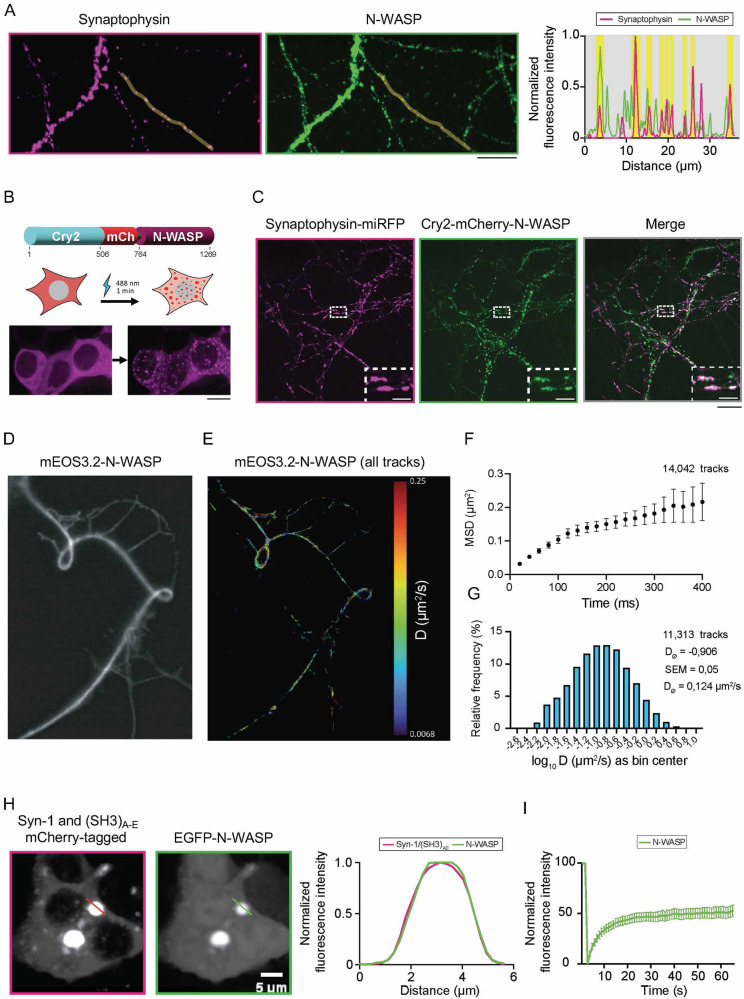
Single-molecule tracking reveals N-WASP behavior in cultured cells. **A** Immunostaining and confocal imaging analysis of synaptophysin and N-WASP in mouse-derived hippocampal neurons. Scale bar = 10 µm. Line profiles on the right show respective colocalization analyses of endogenous synaptophysin (magenta) and N-WASP (green) along the line indicated in representative images. **B** Photoactivation (1 min with 488 nm-laser) of Cry2-mCherry-N-WASP leads to the formation of condensates in cells. Top: scheme of the construct. Bottom: representative images of HEK cells before and after activation. Scale bar = 10 µm. **C** Confocal imaging of rat-derived primary hippocampal neurons 14 days in vitro expressing Cry2-mCherry-N-WASP and Synaptophysin-miRFP. Inset: magnified region indicating the accumulation of N-WASP at the pre-synapses. Scale bars = 20 µm (entire image) and 5 µm (inset). **D** Representative fluorescence image of a neuron expressing mEos3.2-N-WASP. **E** Single-molecule tracks reconstructed for the image shown in 2D after photoconversion of mEos3.2 and color-coded for the measured diffusion coefficients. **F** Mean-square displacement (MSD) curve for 14,042 tracks (from four distinct regions) indicates the confined motion of mEos3.2-N-WASP. **G** Distribution of diffusion coefficients for mEOS3.2-tagged N-WASP from 11,313 tracks analyzed (mobile fraction). The average diffusion coefficient was 0.124 µm^2^ s^−1^. **H** Representative images of HEK293 cells expressing mCherry-Syn-1 with SH3-concatamer of intersectin (SH3)_A-E_, and EGFP-N-WASP. On the right, colocalization analysis of mCherry-Syn-1/(SH3)_A-E_ (magenta) and EGFP-N-WASP (green) along the lines indicated in representative images (left). **I** Fluorescence recovery after photobleaching of EGFP-N-WASP (average ± SEM) from three independent transfections indicates swift fluorescence recovery (~50%).

N-WASP promotes actin remodeling at both pre-and post-synaptic terminals [32]. Using a validated antibody against N-WASP (Supplementary figure S1A-C), we found that N-WASP perfectly co-localized with the SV integral membrane glycoprotein synaptophysin in primary hippocampal neurons (Fig. 2A). Since proteins undergoing LLPS form condensates when fused to oligomerization domains that decrease their saturation concentration (i.e., the minimal concentration at which proteins phase separate), we fused N-WASP to the light-sensitive flavin adenine dinucleotide-binding protein cryptochrome-2 (Cry2) as in previous studies [33, 34]. Optogenetic activation of Cry2-mCherry fused to N-WASP caused rapid cluster formation of the protein in HEK cells (Fig. 2B) in a fashion not normally seen with Cry2-mCherry alone [35]. When we performed live-cell imaging of Cry2-mCherry-N-WASP and Synaptophysin-miRFP in primary hippocampal cell cultures, we further observed that N-WASP co-localized with synaptophysin-positive pre-synaptic compartments (Fig. 2C). To study the dynamic properties of N-WASP in neuronal projections, we fused the photoconvertible fluorophore mEos3.2 to the N-terminus of N-WASP. Upon overexpression in hippocampal neurons, we performed single-particle tracking (Fig. 2D, E) and found that, although confined in an area of around 0.2 µm^2^ (Fig. 2F), N-WASP retained a high motility (*D* = 0.124 µm^2^ s^−1^; Fig. 2G). These data indicate that N-WASP is a highly dynamic molecule that maintains fluid-like properties even in discrete neuronal sub-compartments.

Next, we assessed how N-WASP behaves in the presence of SV condensates by employing a minimal reconstitution system similar to the one used in our previous papers [36, 37]. For this purpose, we co-expressed mCherry-synapsin 1 (Syn-1) and a concatemer of SH3 domains of intersectin also tagged with mCherry (SH3)_A-E_, that readily co-assemble into condensates in mammalian cells [37]. In HEK293 cells, EGFP-N-WASP co-localized with mCherry-Syn-1 within condensates (Fig. 2H). Importantly, N-WASP maintained its high mobility within these condensates, as shown by the rapid fluorescence recovery after laser photobleaching of the GFP-positive droplets (i.e., 50% mobile fraction, *t*_1/2_~8 s) (Fig. 2I). Upon ectopic expression, Syn-1 and α-Syn also co-assembled into condensates [19]. The endogenous N-WASP was also enriched in these condensates in both murine and human cells (Supplementary Fig. S1D–G). Together, these data demonstrate the dynamic behavior of N-WASP in Syn-1/SV condensates in cultured cells.

### N-WASP deficiency influences the formation of α-Syn condensates

**Fig. 3:**
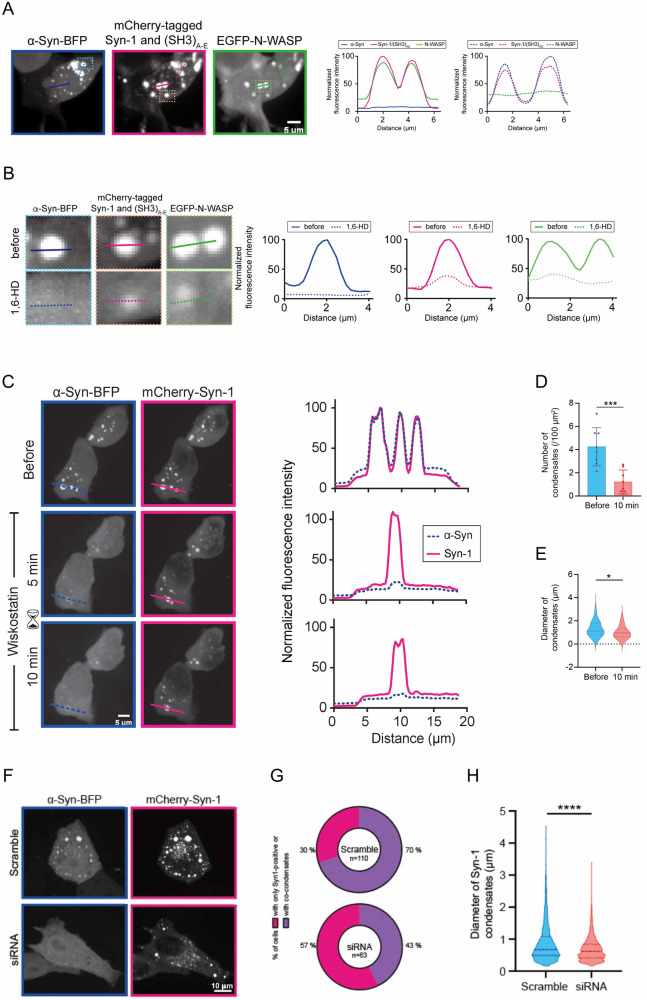
N-WASP influences α-Syn LLPS. **A** Representative images of HEK293 cells expressing α-Syn-BFP, mCherry-Syn-1 with SH3-concatamer of intersectin (SH3)_A-E_ and EGFP-N-WASP. Panels on the right show respective colocalization analyses of α-Syn-BFP (blue), mCherry-Syn-1 (magenta), and EGFP-N-WASP (green) along the lines indicated in representative images. **B** Treatment with 1,6-hexanediol (1,6-HD) leads to dispersion of fluorescence signal from the condensate. Left: condensates before (top) and after (bottom) the addition of 3% 1,6-HD. Right: line profiles over condensates for α-Syn (blue), synapsin condensates (magenta), and N-WASP (green) before (full line) or after 1,6-HD (dashed line) treatment. **C** Wiskostatin treatment (10 μM final concentration) leads to selective dispersion of α-Syn-BFP (α-Syn-BFP) from synapsin condensates (mCherry-Syn-1). On the left, representative images before, 5 min, and 10 min after treatment. On the right, line profiles of the condensates are shown (magenta, syn-1; blue, α-Syn). Number (**D**) and diameter (**E**) of mCherry-Syn-1 condensates condensates decrease upon wiskostatin treatment in a statistically significant fashion (paired *t*-test, two-tailed, confidence level: 95%). **F** Transfection of siRNA against N-WASP reduces the formation of synapsin condensates. Representative images of HeLa cells expressing α-Syn-BFP and mCherry-Syn-1 condensates further transfected with scrambled (control, top) or N-WASP-directed siRNAs (bottom). Scale bar = 10 µm. **G** Fraction of cells containing α-Syn within condensates of Syn-1 decreases strongly upon N-WASP knockdown. **H** The diameters of mCherry-Syn-1 condensates display a statistically significant decrease upon N-WASP knockdown (Kolmogorov–Smirnov test, two-tailed, confidence level: 95%).

In healthy neurons, α-Syn is highly abundant at the synapse [6, 7, 38] and can form liquid-liquid condensates with Syn-1 [19]. To test if N-WASP and α-Syn can be recruited together to synapsin 1-driven condensates, we overexpressed BFP-tagged α-Syn, EGFP-tagged N-WASP, mCherry-tagged Syn-1 and (SH3)_A-E_ in mammalian cells. We observed that Syn-1/(SH3)_A-E_ condensates that contained N-WASP lacked α-Syn and, conversely, distinct condensates harboring α-Syn largely lacked N-WASP (Fig. 3A). Next, we assessed whether N-WASP-containing structures were indeed condensates rather than aggregate-like inclusions. To achieve this, cells were treated with the aliphatic alcohol 1,6-hexanediol, which broadly disrupts hydrophobic interactions present in condensates [39]. Live-cell imaging experiments showed little evidence of aggregate formation, since treatment with 1,6-HD could largely abolish condensates (Fig. 3B). Building on our observations that α-Syn and N-WASP did not always co-condense in the same structures, we tested whether N-WASP inhibition could influence the sequestering of α-Syn into Syn-1 condensates. As a first proof-of-principle experiment, we incubated cells with the carbazole derivative wiskostatin which is capable of binding and inhibiting N-WASP [40]. In wiskostatin-treated cells, we observed a marked reduction in the number and size of condensates, along with the depletion of α-Syn from the remaining condensates (Fig. 3C–E). To confirm these observations, we downregulated N-WASP using short interfering RNA (siRNA) and found that diminished N-WASP expression led to a reduction in the fraction and size of Syn-1 condensates that were positive for α-Syn (Fig. 3F–H). Together, these data suggest that N-WASP can influence α-Syn recruitment into Syn-1-containing condensates.

### WSP-1/N-WASP expression modulates α-Syn pathology in *C. elegans*

**Fig. 4:**
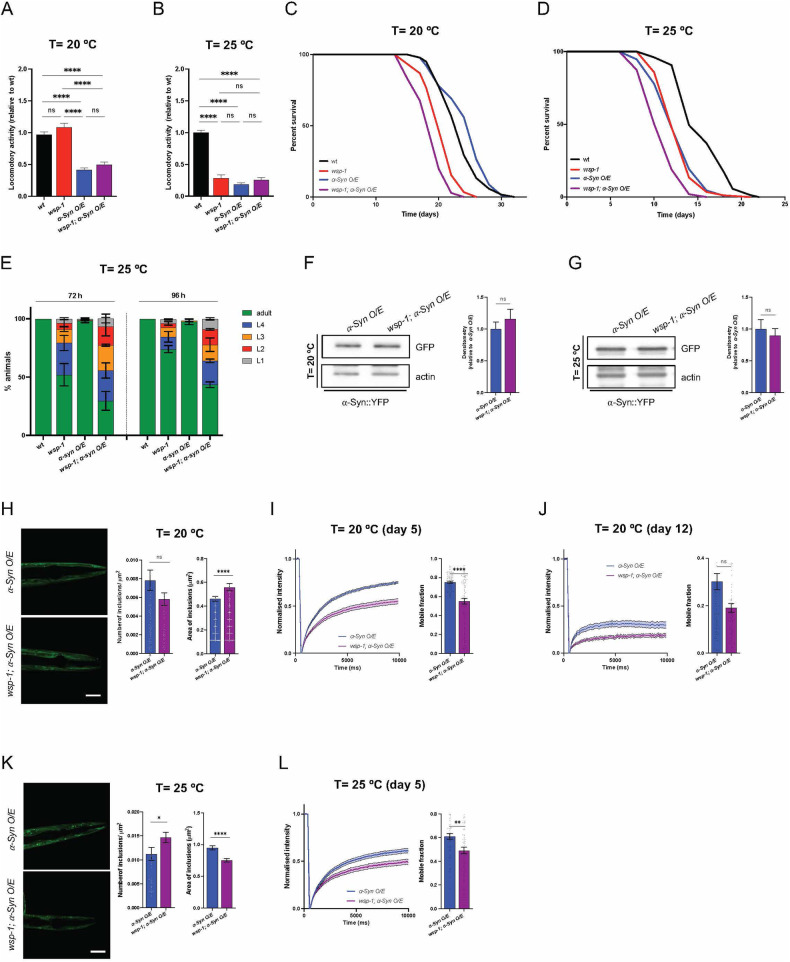
*wsp-1(lof)* negatively affects the dynamics of α-Syn-containing condensates in *C. elegans.* Locomotor activity of **A** 7-day-old and **B** 4-day-old wt (black), *wsp-1(lof)* (red), *α-Syn O/E* (blue), and *wsp-1(lof);a-Syn O/E* (purple) animals grown at **A** 20 °C or **B** 25 °C. Each bar represents mean±SEM (One-way ANOVA with Tukey’s multiple comparison test, ns not significant, ****p < 0.0001; number of animals = 24 (**A**) and 16-36 (**B**) from 3 (**A**) and 5 (**B**) experiments). **C**, **D** Representative lifespan assays of wt, *wsp-1(lof)*, *α-Syn O/E*, and *wsp-1(lof);α-Syn O/E* animals grown at the indicated temperatures. **E** Developmental assay at 25 °C. The percentage of the population at the indicated larval stages at 72 h (left panel) and 96 h (right panel) after hatching. Bars represent mean±SEM. Representative western blots (left) and densitometry (right) of α-Syn::YFP protein expression, in 5-day-old *α-Syn O/E* and *wsp-1(lof);α-Syn O/E* animals grown (**F**) at 20 ^o^C and (**G**) 25 °C. Bars represent mean ± SEM (Unpaired *t*-test, ns not significant, *n* = 3). **H** Representative images and quantification of the number and size of α-Syn::YFP inclusions in the heads of 10-day-old *α-Syn O/E* and *wsp-1(lof);α-Syn O/E* animals grown at 20 °C. Scale bar = 40 μm. Bars represent mean±SEM (Mann–Whitney test, ns not significant, ****p < 0.0001, *n* = 61–72 animals from 4 independent experiments). FRAP traces (left) and quantification of mobile fraction (right) of individual α-Syn::YFP inclusions in *α-Syn O/E* and *wsp-1(lof);α-Syn O/E* animals grown for **I** 5 and **J** 12 days at 20 °C. Lines represent mean normalized intensity±SEM and bars represent mean ± SEM (Mann–Whitney test, ns not significant, ****p < 0.0001; **I** *n* = 94–95 inclusions, *n* = 3 biological replicates; **J** *n* = 92–100 inclusions, *n* = 3 biological replicates). **K** Representative images of 5-day-old nematodes grown at 25 °C (scale bar = 40 μm). Quantification of inclusion number and size is reported as mean±SEM (Mann–Whitney test, *p < 0.05, ****p < 0.0001, n = 45-50 inclusions, *n* = 3 biological replicates). **L** FRAP traces (left) and quantification of mobile fraction (right) of individual α-Syn::YFP inclusions in 5-day-old *α-Syn O/E* and *wsp-1(lof);α-Syn O/E* animals grown at 25 °C. Lines and bars represent mean±SEM (Mann–Whitney test, **p < 0.01, *n* = 68–75 inclusions, *n* = 3 biological replicates).

To investigate if N-WASP can influence α-Syn homeostasis in vivo, we employed *C. elegans* strains carrying the transgene *pkIs2386[unc-54p*::*α-Syn:YFP]* and expressing YFP-tagged α-Syn in the body wall muscle cells. As a well-established model for studying proteotoxicity in vivo in nematodes [41], we initially assessed locomotion at standard experimental conditions (20 °C) and found that *wsp-1(lof)* mutants showed no obvious differences compared to wt animals, whereas α-Syn O/E nematodes exhibited locomotor defects (Fig. 4A). Based on our thrashing assays, the genetic inhibition of WSP-1 protein expression by *wsp-1(lof)* allele [42] did not worsen the phenotype of α-Syn O/E nematodes (Fig. 4A). We also grew animals at a warmer temperature starting from hatching, since it is known that at 25 °C nematodes exhibit faster development, reduced lifespan and changes in proteostasis [43, 44]. Compared to wt animals, *wps-1(lof)*, *α-Syn O/E* and *wps-1(lof);α-Syn O/E* equally compromised *C. elegans* locomotion at 25 °C (Fig. 4B), suggesting that WSP-1 deficiency had a minor effect on thrashing defects due to α-Syn O/E. However, while α-Syn O/E had a marginal lifespan effect at 20 °C, it negatively impacted *C. elegans* survival at 25 °C (Fig. 4C, D and Supplementary table ST4). In each experiment, *wsp-1(lof)* compromised *C. elegans* survival at both temperatures and had an additive negative effect on α-Syn O/E lifespan at higher temperatures (Fig. 4C, D and Supplementary Table ST4). Statistical analysis of median lifespan suggested that *wsp-1(lof)* and α-Syn O/E may act independently on organismal survival (Supplementary Table ST4). Of note, α-Syn O/E had a negligible developmental effect in short-lived *wsp-1(lof)* animals that were grown at warmer temperature (Fig. 4E).

Having assessed a few phenotypes linked to α-Syn O/E and *wsp-1(lof)* in *C. elegans*, we sought to test whether WSP-1 deficiency could shift the balance of α-Syn inclusions from liquid-like condensates to more toxic species, knowing that over time α-Syn inclusions convert into intracellular amyloid-like aggregates in the body wall muscles of *C. elegans* [9]. We performed western blot analysis and showed that *wsp-1(lof)* did not affect the expression of the transgene encoding YFP-tagged α-Syn neither at 20 °C nor at 25 °C (Fig. 4F, G). We quantified the number and size of α-Syn inclusions in nematodes grown at 20 °C and found that *wsp-1(lof)* promoted the formation of larger α-Syn-containing inclusions compared to those in control animals (Fig. 4H). We performed FRAP analysis in young and relatively old (12 days after hatching) animals and found that *wsp-1(lof)* negatively affected α-Syn::YFP mobility in *C. elegans* muscle cells (Fig. 4I, J). At a warmer temperature, adult *wsp-1(lof)* animals showed an increased number of smaller α-Syn::YFP inclusions compared to controls (Fig. 4K). Importantly, genetic inhibition of WSP-1 protein expression reduced α-Syn::YFP mobility compared to control nematodes (Fig. 4L). Together, our data suggest that WSP-1/N-WASP deficiency can diminish the mobile fraction of α-Syn::YFP within inclusions and may possibly promote α-Syn conversion into amyloid-like hydrogels.

In PD pathology, α-Syn readily forms insoluble fibrils that propagate among neurons and surrounding glia [45]. To assess the burden caused by preformed fibrils (PFFs) of α-Syn in N-WASP deficient cells, we employed induced-pluripotent stem cell (iPSC)-derived dopaminergic neurons and found that N-WASP downregulation further undermined the maintenance of neurite branches upon exposure to exogenous α-Syn PFFs (Supplementary Fig. 2A, B), suggesting that N-WASP dysfunction may promote α-Syn pathology.

### Inhibition of mitochondrial complex I does not phenocopy *wsp-1(lof)* effects on α-Syn pathology

**Fig. 5:**
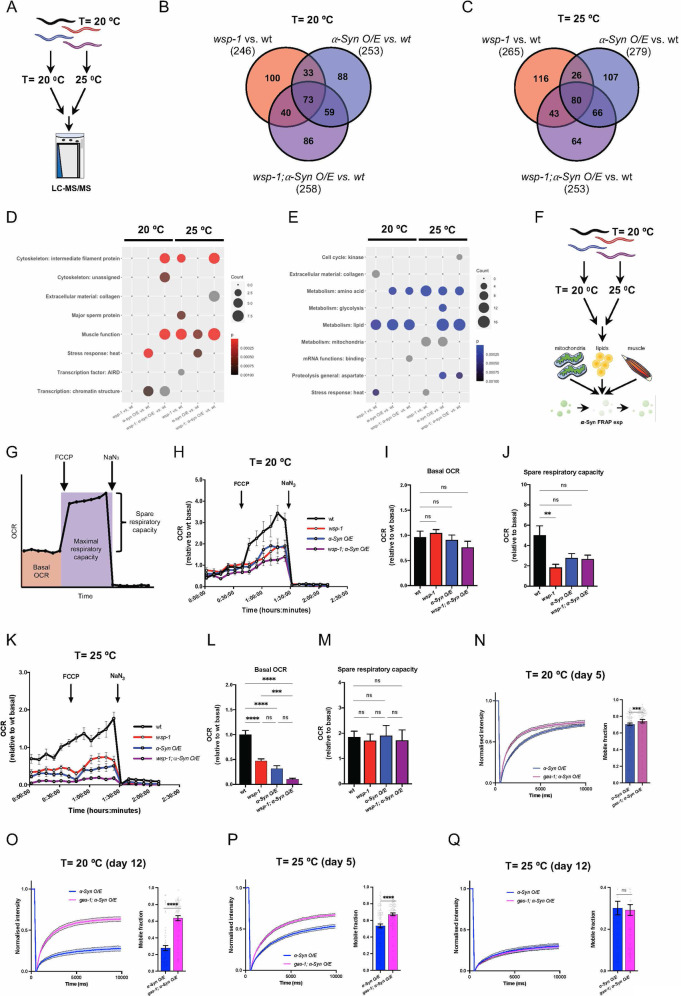
Mitochondrial complex I deficiency stimulates α-Syn::YFP LLPS. **A** Schematic representation of the LC-MS/MS experiment. Venn diagrams indicating the number of differentially expressed proteins (relative to wt) in *wsp-1(lof)*, *α-Syn O/E*, and *wsp-1(lof);α-Syn O/E* animals grown at **B** 20 ^o^C and **C** 25 °C. Bubble plots showing the significant WormCat categories that were **D** upregulated or **E** downregulated in the indicated comparisons. Bubble size indicates the number of differentially expressed proteins in the corresponding category, and bubble color corresponds to *p* value. **F** Schematic summary of the experiments performed using wt and mutant nematodes grown at 20 °C or 25 °C. **G** Schematic representation of an OCR trace and how basal respiration and maximal respiratory capacity were calculated. **H** OCR traces of 5-day-old animals grown at 20 °C. Each point reports mean±SEM across replicates (*n* = 15–19 from 4 experiments). Quantification of **I** basal OCR and **J** spare respiratory capacity in 5-day-old animals grown at 20 °C. Bars represent mean ± SEM (Kruskal–Wallis test with Dunn’s multiple comparison test, ns not significant, **p < 0.01, *n* = 15–19 from 4 independent experiments). **K** Representative OCR traces of 5-day-old animals grown at 25 °C. Each point reports mean±SEM across replicates (*n* = 9–12 from 3 independent experiments). Quantification of **L** basal OCR and **M** spare respiratory capacity of 5-day-old wt animals grown at 25 °C. Bars represent mean±SEM (One-way ANOVA with Tukey’s multiple comparison test, ns not significant, ***p < 0.001, ****p < 0.0001, *n* = 9–12 from 3 experiments). FRAP of α-Syn::YFP inclusions in the head region of (**N**, **P**) 5-day- and (**O**, **Q**) 12-day-old animals grown at **N**, **O** 20 °C or **P**, **Q** 25 ^o^C. Left, normalized recovery curves of *α- Syn O/E* (blue) and *gas-1;α-Syn O/E* (pink). Right, quantification of the mobile fraction of individual inclusions. Lines and bars represent mean ± SEM (Mann–Whitney test, ns not significant, ***p < 0.001, ****p < 0.0001, number of inclusions = 94–95 (**N**), 70–71 (**O**), 95–100 (**P**), 66–68 (**Q**), from 3 biological replicates).

To better understand how WSP-1/N-WASP modulates α-Syn phase transition in *C. elegans*, we decided to perform additional LC-MS/MS experiments on animals grown at 20 °C and 25 °C (Fig. 5A). Compared to wt animals grown at 20 °C, we identified 246, 253, and 258 differentially expressed proteins in *wsp-1(lof), α-Syn O/E*, and *wsp-1(lof);α-Syn O/E*, respectively (Fig. 5B and Supplementary table ST5). At 25 °C, we obtained 265, 279, and 253 differentially expressed proteins in *wsp-1(lof), α-Syn O/E*, and *wsp-1(lof);α-Syn O/E*, respectively (Fig. 5C and Supplementary table ST5). All these three mutant strains displayed an upregulation of proteins related to muscle function among the most obvious changes (Fig. 5D and Supplementary table ST6). Moreover, many proteins involved in mitochondrial metabolism were downregulated in *wsp-1(lof)* and *α-Syn O/E* compared to wt at 25 °C. We observed depletion of proteins involved in metabolism, including lipid and amino acid metabolism, that were more prominent in *α-Syn O/E*, and *wsp-1(lof);α-Syn O/E* compared to *wsp-1(lof)* animals grown at 25 °C (Fig. 5E and Supplementary table ST6). Together, our data suggest that aberrant metabolism may accompany the pathology due to α-Syn O/E and WSP-1/N-WASP deficiency.

To validate some of these *in-silico* predictions, we decided to genetically manipulate a few metabolic pathways in nematodes grown at the two different temperatures (Fig. 5F). Since aberrant mitochondrial activity is associated with many synucleinopathies [4, 46], we measured oxygen consumption rate (OCR) using a Seahorse Analyzer (Fig. 5G). At normal growing conditions (20 °C), α-Syn O/E animals had the tendency to respire less than wt, with a basal OCR and spare respiratory capacity similar to those detected in *wsp-1(lof);α-Syn O/E* animals (Fig. 5H–J). Interestingly, animals carrying a *wsp-1(lof)* showed a reduced spare respiratory capacity compared to wt animals (Fig. 5J). Notably, α-Syn O/E further exacerbated the mitochondrial respiratory defects of *wsp-1(lof)* mutant animals when grown at 25 °C (Fig. 5K–M). Together, these data suggest that WSP-1/N-WASP deficiency impairs mitochondrial respiration, with α-Syn O/E having an additive effect only in certain conditions. To determine if inhibition of mitochondrial bioenergetics may phenocopy the effect of *wsp-1(lof)* on α-Syn phase transition, we employed *gas-1(fc21)* mutant nematodes, which also exhibit reduced mitochondrial respiration and shortened lifespan compared to wildtype as shown in our previous studies [47–49]. Surprisingly, FRAP experiments at different ages and temperatures showed that α-Syn::YFP was relatively more mobile in *gas-1* mutants compared to control nematodes (Fig. 5N-Q). At least in *C. elegans*, mitochondrial inhibition does not recapitulate the changes of α-Syn mobility caused by WSP-1 deficiency, possibly indicating that WSP-1 influences α-Syn pathology independently of its effects on mitochondrial bioenergetics.

**Fig. 6:**
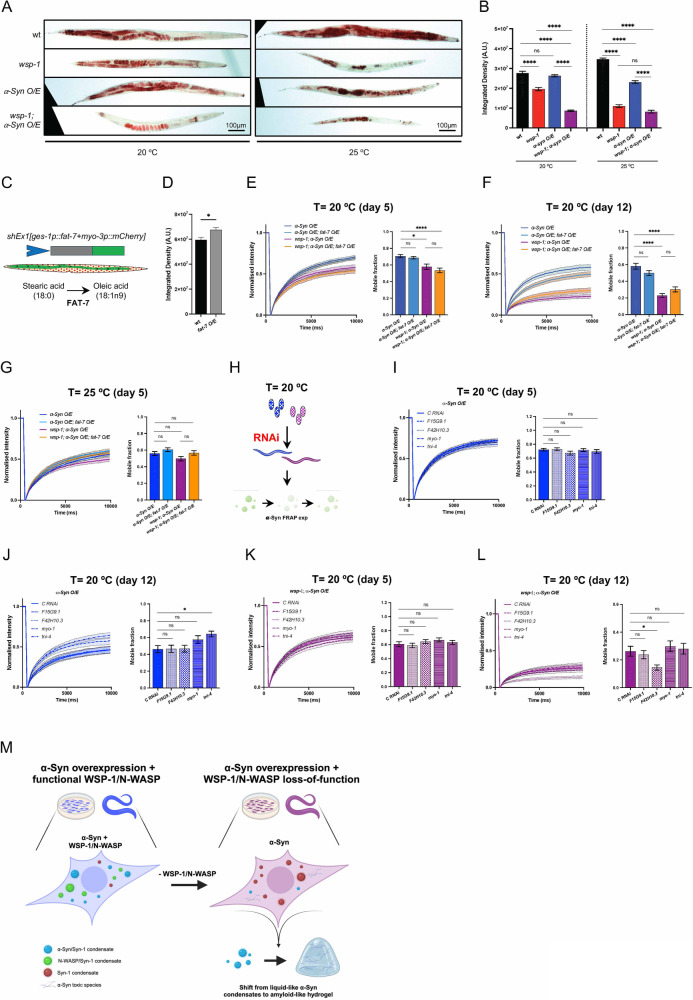
Changes in lipid content and muscle function accompany α-Syn::YFP LLPS. Representative **A** Oil Red O staining (scale bar = 100 µm) and **B** its quantification in wt, *wsp-1(lof)*, *α -Syn O/E*, and *wsp-1(lof);α-Syn O/E* animals grown at 20 °C or 25 °C. Each bar represents mean±SEM (Kruskal–Wallis test with Dunn’s multiple comparison test, ns not significant, ****p < 0.0001, n = 50–60 animals from 3 independent experiments). **C** Schematic representation of the *shEx1[ges-1p::fat-7+myo-3p::mCherry]* transgene expressing FAT-7 in *C. elegans* intestine. FAT-7 desaturates stearic acid into oleic acid. **D** Quantification of ORO staining in wt and *fat-7 O/E* animals grown at 20 °C. Each bar represents mean ± SEM (Student’s t-test, *p < 0.05, *n* = 36 animals from 3 independent experiments). (Fluorescent recovery after photobleaching of α-Syn::YFP inclusions in the head region of **E**, **G** 5-day- and **F** 12-day-old animals grown at **E**, **F** 20 °C and **G** 25 °C. Left: normalized recovery curves of *α-Syn O/E* (blue), *α-Syn O/E;fat-7 O/E* (light blue), *wsp-1(lof);α-Syn O/E* (purple), and *wsp-1(lof);α-Syn O/E; fat-7 O/E (*orange). Right: quantification of the mobile fraction of individual inclusions. Lines and bars represent the mean±SEM (Kruskal–Wallis with Dunn’s multiple comparison test, ns not significant, *p < 0.05, ****p < 0.0001, number of inclusions = 54–63 (**E**), 62–78 (**F**), 58–61 (**G**), *n* = 3 biological replicates). **H** Schematic summary of FRAP experiments performed using *α-Syn O/E* and *wsp-1(lof);α-Syn O/E* nematodes grown on RNAi at 20 °C. Fluorescent recovery after photobleaching of α-Syn::YFP inclusions in the head region of animals grown at 20 °C. On the left, normalized recovery curves are reported for **I**, **J** *α-Syn O/E* or **K**, **L** *wsp-1(lof);α-Syn O/E* animals grown on RNAi against control, *F15G9.1, F42H10.3, myo-1, tni-4*. On the right of each panel, quantification of the mobile fraction of individual inclusions are reported. Lines and bars represent the mean±SEM (Kruskal-Wallis with Dunn’s multiple comparison test, ns none-significant, *p < 0.05, number of inclusions = 45–48 (**I**), 36-47 (**J**), 4748 (**K**), 47-48 (**L**), *n* = 3 biological replicates). **M** Schematic summary of our findings. The expression of Syn-1 promotes the formation of intracellular condensates that can recruit either α-Syn or N-WASP. Loss of WSP-1/N-WASP negatively affects the recruitment of α-Syn to Syn-1-containing condensates, possibly shifting the propensity of α-Syn to form amyloid-like hydrogel. Created with BioRender.com.

Next, we assessed neutral lipids and carried out Oil Red O (ORO) staining in our four strains. We found that *wsp-1(lof)* and *wsp-1(lof);α-Syn O/E* animals had a reduced ORO staining at both 20 °C and 25 °C compared to wt, whereas *α-Syn O/E* animals showed lower signal only at 25 °C (Fig. 6A, B). To alter lipid metabolism in *C. elegans*, we used an extrachromosomal array overexpressing FAT-7 (Fig. 6C), a fatty acid desaturase that converts stearic acid into oleic acid and promotes *C. elegans* lifespan extension [50]. Although FAT-7 was expressed only in the intestine, it could increase the overall lipid content in the transgenic nematodes compared to wt ones (Fig. 6D). We carried out FRAP experiments in α-Syn::YFP O/E animals and found that FAT-7 expression in the gut was not sufficient to modify the mobility of α-Syn::YFP condensates in the muscle cells of neither control nor *wsp-1(lof)* mutant nematodes (Fig. 6E–G). This dataset suggests that changes in lipid metabolism may not be the main driver of α-Syn liquid phase transition.

Finally, we tested whether proteins related to muscle function may modulate condensates in animals grown at 20 °C, given that this term was upregulated in *wsp-1(lof);α-Syn O/E* animals at this temperature (Fig. 5D and Supplementary table ST6). Using a validated RNAi feeding protocol starting from hatching as described in our previous studies [47, 48, 51], we downregulated *F15G9.1, F42H10.3, myo-1*, and *tni-4* genes in *α-Syn O/E* and *wsp-1(lof);α-Syn O/E* animals (Fig. 6H). Our RNAi experiments showed negligible changes in FRAP of α-Syn::YFP liquid-like droplets in young as well as older adult animals (Fig. 6I, J). Similarly, there was no rescue of condensate mobility following downregulation of the candidates in *wsp-1(lof);α-Syn O/E* animals, neither in younger animals nor in older animals (Fig. 6I–L). In summary, our data suggest that some of the proteomic changes may contribute to the pathological phenotype of WSP-1/N-WASP deficiency, however they are not the main cause of α-Syn LLPS and its eventual transition into amyloid-like hydrogel (Fig. 6M).

## Discussion

Synucleinopathies are common age-related neurodegenerative diseases, with PD accounting for the high incidence in people aged 80 years or older [52, 53]. Despite the advance of knowledge in the scientific community, our understanding of α-Syn interactome changes in certain biological contexts (e.g., aging) as well as upon exposure to environmental cues remains very limited. Similarly, it remains elusive which molecular mechanisms govern the liquid phase transition and aggregation of α-Syn.

Here, we investigate the interactome of α-Syn and report new evidence connecting α-Syn with cytoskeleton components. Our findings are in line with previous studies showing that wild type and mutant α-Syn can interact with actin [54] and actin-binding proteins such as gelsolin or spectrin [54–56]. Consistent with prior evidence, α-Syn interaction with actin and actin-binding proteins includes dysregulation of actin dynamics, altered exo- and endocytosis, and reduced mitochondrial function [54, 56]. In addition to α-Syn-dependent regulation of the actin cytoskeleton, other PD-related proteins, such as Parkin/PARK2 [57, 58] and VPS35/PARK17 [59], have also been shown to influence the remodeling of the actin cytoskeleton. In an attempt to elucidate mechanistically the biological meaning of this network of interactions, we show that N-WASP expression and activity can modify the recruitment of α-Syn into liquid-like condensates in cultured cells. Consistent with a role of N-WASP in modulating α-Syn recruitment into intracellular inclusions, we provide a first line of evidence demonstrating that WSP-1 deficiency can promote α-Syn pathology in *C. elegans*. Indeed, when we exposed nematodes to different experimental paradigms, we found that α-Syn O/E is relatively well tolerated and does not have a negative impact on development. However, α-Syn seems to become more toxic in WSP-1 deficient cells, possibly because it converts into less mobile condensates that, over time, may transform into pathogenic species. This in vivo evidence suggests that disease-causing *WASL* mutations may define the threshold of susceptibility to α-Syn expression, thereby predisposing an organism to α-Syn proteotoxicity. Consistent with the complex etiology and epidemiology of PD [60], it may be that N-WASP loss of function variants represent one of the genetic predispositions promoting α-Syn pathology under stress and environmental challenges. Although further studies are necessary, our findings indicate a direct relationship between α-Syn expression and N-WASP deficiency, which may partially explain the causative genetic link of compound heterozygous *WASL* mutations with an early onset familial form of PD [30].

While the role of the cytoskeleton is emerging as an important player in synucleinopathies, a vast literature has reported genetic mutations linking mitochondria with parkinsonism and/or inherited PD [4, 61–63]. Given these lines of evidence, we investigated the impact of *wsp-1(lof)* on mitochondria in *C. elegans*. We report that genetic inhibition of *wsp-1/WASL* can impair mitochondrial respiration and worsen metabolic defects due to α-Syn overexpression. Although we could not conclusively elucidate the molecular mechanisms underlying these observations, our findings recapitulate some of the mitochondrial defects that are often associated with synucleinopathies. At least in nematodes, it seems that aberrant mitochondrial respiration may be exacerbated by the cumulative effects of WSP-1/N-WASP deficiency and α-Syn overexpression. Despite the influence of *wsp-1(lof)* on mitochondrial respiration, complex I deficiency alone was not sufficient to recapitulate the α-Syn::YFP phase transition changes observed in *wsp-1(lof)* animals. Rather than through metabolic changes as upstream effects, we speculate that WSP-1/N-WASP deficiency may prime the susceptibility of the proteome to additional stress, which may further shift the proteostatic balance of α-Syn, its propensity to undergo LLPS and eventually its toxic aggregation. In addition to the proteome collapse, cells may undergo further metabolic challenges due to aberrant mitochondrial bioenergetics as a consequence of N-WASP deficiency. To support this scenario, it has been shown that aberrant expression of the WAS family member WASp causes mitochondrial network fragmentation and loss of mitochondrial respiration in haemopoietic cells [64]. As in our experimental paradigms, the loss of a WAS family member may critically affect the threshold at which cells can buffer stress, since defective cytoskeleton remodeling may compromise mitochondrial dynamics, trafficking and biogenesis, with obvious consequences on cellular homeostasis and survival.

Taken together, our cross-species study broadens the molecular landscape of the α-Syn interactome and offers a first experimental framework to better understand how actin remodelers may contribute to α-Syn phase transition into toxic species.

## Experimental procedures

### Antibodies

The following primary antibodies were used in this study: mouse anti-alpha-synuclein (Bio Legend, 4B12/Synuclein, 807801), mouse anti-alpha-synuclein (Invitrogen, AHB0261), mouse anti-GFP (Roche, 11814460001), mouse anti-HA (Sigma, HA-7, H9658), rabbit anti-HA (Sigma, H6908), rabbit anti-N-WASP (Cell Signaling, 30D10, #4848), rabbit anti-N-WASP (polyclonal raised against peptide 385–401) [65], rabbit anti-N-WASP (Invitrogen, PA5-52198), mouse anti-synaptophysin 1 (SySy, 7.2, 101 0011), mouse anti-actin (Abcam, ab14128). Secondary antibodies used in this study were: HRP-conjugated anti-mouse secondary (Thermo Fisher Scientific), HRP-conjugated anti-rabbit secondary (Promega, W4011), Alexa Fluor™ 488-conjugated goat anti-mouse IgG (Invitrogen, A-11001), Alexa Fluor™ 568-conjugated goat anti-rabbit IgG (Invitrogen, A-11011) and Abberior STAR 488-conjugated goat anti-rabbit IgG (Abberior).

### *Caenorhabditis elegans* strains

The following strains were used in this study: wild type N2 (Bristol), BAN419 *gas-1(fc21)X;pkIs2386[unc-54p::α-syn::YFP+unc-119(*+*)]*, BAN534 *wsp-1(gm324)IV*, BAN550 *wsp-1(gm324)IV;pkIs2386[unc-54p::α-syn::YFP+unc-119(*+*)]*, BAN686 *shEx34[myo-3p::mCherry]*, BAN688 *pkIs2386[unc-54p::alpha synuclein::YFP + unc-119(+)];shEx34[myo-3p::mCherry]*, BAN689 *wsp-1(gm324)IV;pkIs2386[unc-54p::alpha synuclein::YFP + unc-119(+)];shEx34[myo-3p::mCherry]*, BAN690 *shEx1[ges-1p::fat-7 + myo-3p::mCherry]*, BAN692 *pkIs2386[unc-54p::alpha synuclein::YFP + unc-119(+)];shEx1[ges-1p::fat-7 + myo-3p::mCherry]*, BAN693 *wsp-1(gm324)IV;pkIs2386[unc-54p::alpha synuclein::YFP + unc-119(+)];shEx1[ges-1p::fat-7 + myo-3p::mCherry]*, NL5901 *pkIs2386[unc-54p::α-syn::YFP+unc-119(+)]*. Nematodes were maintained following standard culture methods as in some of our previous studies [47, 48, 51]. All RNAi experiments were performed at indicated temperatures by feeding nematodes with HT115 *E. coli* expressing dsRNA against target genes. Some strains were provided by the CGC, which is funded by the NIH Office of Research Infrastructure Programs (P40 OD010440).

### Cell cultures

HeLa cells and human embryonic kidney HEK293 and HEK293T cells were grown in DMEM (Gibco) supplemented with 10% fetal bovine serum and 1% penicillin/streptomycin (100 U/ml penicillin; 100 mg/ml streptomycin). Cells were transfected with 2 µg of plasmid DNA using either Lipofectamine 2000 (Thermo Fisher Scientific) or Turbofectin (Origene) following the manufacturer’s instructions. For experiments in HeLa cells downregulating N-WASP, the siRNA (siRNA ID: 137396; Thermo Fisher Scientific) was transfected with Lipofectamine RNAiMAX (Thermo FisherScientific), 24 h prior to plasmid transfection with Lipofectamine 2000 for the transient protein expression. For co-IP mass spectrometry sample preparation, suspension cultures of Expi293F™ cells (HEK derivative) were used. Cultures were maintained in an Expi expression medium as described in the manufacturer’s protocol (8% CO_2_, 37 °C, 125 rpm). For protein expression, suspension cultures (30 ml) were transfected with 30 µg of plasmid DNA following the ExpiFectamine transfection guidelines.

B16-F1 cells were routinely grown in Dulbecco’s Modified Eagles Medium (DMEM, Gibco) supplemented with 10% FBS and 2 mM l-glutamine (Gibco) at 37 °C and in 5% CO_2_ atmosphere. CRISPR/Cas9-mediated disruption in B16-F1 cells of the *Wasl* encoding N-WASP was achieved essentially following standard procedures [66] and transfection of a vector harboring a CRISPR-gRNA sequence targeting exon 1 (5′-GAGAGACTCGTTCTCTTGCG-3′). Monoclonal cell lines were expanded from single-cell clones followed by screening for the absence of N-WASP expression by Western blotting (not shown). Selected clones devoid of N-WASP expression (Supplementary figure S1A) were confirmed to lack any *Wasl* wildtype allele by genotyping, as follows: sequences resulting from PCR amplification of the *Wasl* targeting region potentially harboring the site of disruption were subjected to Sanger sequencing and analysis by TIDE sequence trace decomposition [67]. N-WASP expression was also assessed by western blot using a previously characterized triple KO cell line lacking Sra1, PIR121/CYFIP, and N-WASP [65] (Supplementary Fig. S1A).

Primary hippocampal neurons were prepared from P0 wild-type mice (C57BL6/J) as previously described [19]. Neurons were seeded on coverslips and transfected using a standard calcium phosphate protocol. For single molecule tracking experiments, primary rat hippocampal neurons were used.

Induced-pluripotent stem cell (iPSC)-derived midbrain dopaminergic neurons (mDANs) were generated from smNPCs, as previously described [68, 69]. Briefly, iPSC cultures were dissociated using accutase, centrifuged and resuspended in neural induction medium (DMEM-F12 (Invitrogen) 1:200 N2 supplement (Invitrogen), 1:100 B27 supplement without vitamin A (Invitrogen), 2 mM of GlutaMax, 10 μM of SB‐431542, 1 μM of dorsomorphin, 0.5 μM of purmorphamine, and 3 μM CHIR99021), supplemented with 10 μM of ROCK inhibitor (Tocris). Cells were cultured in nonadherent plates for 3 days, and on days 4–5, medium was changed to smNPC maintenance medium (neural induction medium lacking SB‐431542 and dorsomorphin, with 150 μM of ascorbic acid added). On day 6, embryoid bodies were triturated by pipetting and plated into Matrigel‐coated 12‐well plates. Cells were split using accutase and passaged for at least 6 splits to remove non-smNPC cells from the cultures. Subsequent differentiation into mDANs was initiated approximately 2 days after the previous passage. Medium was changed to mDAN induction medium (DMEM-F12 (Invitrogen) 1:200 N2 supplement (Invitrogen), 1:100 B27 supplement without vitamin A (Invitrogen), 100 ng/ml FGF8 (Invitrogen), 1 µM purmorphamine, and 200 µM Ascorbic Acid), and changed every 2–3 days. After 8 days, medium was changed to mDAN maturation medium (DMEM-F12 (Invitrogen) 1:200 N2 supplement (Invitrogen), 1:100 B27 supplement without vitamin A (Invitrogen), 10 ng/ml BDNF (PeproTech), 10 ng/ml GDNF (Peprotech), 1 ng/ml TGF-b3, 200 μM Ascorbic Acid, and 500 μM dbcAMP (Sigma)). On days 8-10, maturation medium was supplemented with 0.5 µM purmorphamine. On day 9, cultured cells were split 1:3 into small clumps using accutase. Cells were maintained in mDAN maturation medium until day 21. Transfection of shRNA (VectorBuilder, ID VB010000-9346qaz and VB900072-7764yby) was performed at day 10 with Lipofectamine 3000 (Invitrogen), and medium was changed the following day. For mDAN treatment, recombinant human α-synuclein (A140C) was purified from bacteria and labeled with Alexa Fluor™ 647 C_2_ Maleimide (Thermo Fisher Scientific) as previously described [19]. The preformed fibrils were prepared by mixing 10:1 (unlabeled-to-labeled) α-synuclein, as previously described [70]. Freshly sonicated α-Syn-Alexa Fluor 647 PFFs (100 nM final concentration) were added on day 14. On day 21, mDANs were fixed with warm 4% PFA/4% sucrose.

### Co-immunoprecipitation for mass spectrometry analysis

Expi293 cells expressing either HA-tagged α-synuclein or mock controls were harvested 3 days after transfection. Cells were lysed in a buffer that contained 25 mM Tris-HCl pH 7.4, 150 mM NaCl, and 0.5 mM TCEP (buffer A) supplemented with complete EDTA-free Protease Inhibitor Cocktail (Roche) by three consecutive cycles of freezing and thawing. Soluble protein was separated from cellular debris by centrifugation at 20,000 × *g* at 4 °C for 45 min. Approximately 2000 µg of soluble protein supernatant was subjected to 100 µl of pre-equilibrated anti-HA agarose beads (Thermo Fisher Scientific, 26181) in a Poly-Prep® gravity flow chromatography column (Biorad) at 4 °C for 1.5 h with slow rotating agitation. Then, beads were washed with 10 ml of wash buffer (buffer A), and bound proteins were eluted with hot, non-reducing 2x SDS-PAGE loading buffer (125 mM Tris-HCl pH 6.8, 20% glycerol, 4% SDS, and 0.1% bromophenol blue).

### Co-immunoprecipitations and western blot analysis

Co-IPs were performed using Immunoprecipitation kit (Abcam; catalog no.: ab206996) following the manufacturer’s instructions. Briefly, cells expressing HA-α-Syn or HA-YFP (48 h after transfection) were washed once with PBS and incubated with 2 mM dithiobis (succinimidyl propionate) (Thermo Fisher Scientific, PG82081) for 30 min for reversible crosslinking. The crosslinking reaction was stopped by incubating the cells with 25 mM Tris for 15 min. Cells were then washed with PBS and harvested in 500 μl cold nondenaturing lysis buffer (Abcam, ab206996). Cells were lysed at 4 °C for 30 min on a rotatory mixer. Lysates were cleared by centrifugation at 10,000 × *g* for 10 min, and protein concentration was determined using a Bradford assay. Approximately 500 μg of total protein was incubated with an anti-HA antibody (1:100) overnight at 4 °C on a rotatory mixer. Then, 40 μl of A/G Sepharose bead slurry was added to the protein–antibody mix and incubated for 1 h at 4 °C. Beads were then collected and washed by slow-speed centrifugation. Bound proteins were eluted from the beads by adding 40 μl 2× SDS-PAGE loading buffer (125 mM Tris-HCl pH 6.8, 4% SDS, 20% glycerol, 10% 2-mercaptoethanol, and 0.005% bromophenol blue) and boiling for 5 min.

Samples were resolved on 12% poly-acrylamide gels and transferred onto nitrocellulose membranes using semi-dry transfer Trans-Blot Turbo (Bio-Rad). Protein detection was performed using the following primary antibodies at a dilution of 1:1000, followed by HRP-conjugated secondary antibodies at a 1:2000 dilution. Immunoblots were developed in ECL and imaged using Chemidoc imaging system (Bio-Rad).

### Confocal analysis and FRAP in vivo

Nematodes were transferred into a drop of 20 mM levamisole on a glass slide with a pad of 2% agarose, and a glass coverslip was gently placed on top. Animals were imaged for a maximum of 20 min following mounting on the slide. Z-stack images of alpha-synuclein inclusions were acquired from the head region, using a 20× objective and a Zeiss LSM900 confocal microscope. Inclusion size and number were assessed on maximum Z projections using ImageJ.

For FRAP analysis, animals were imaged using a 20× objective with 4× crop. Individual inclusions in the head to the animal were selected. Following image acquisition of 5 frames (<120 ms per frame), inclusions were photobleached, and subsequent fluorescent recovery was assessed for a further 95 frames. Intensity measurements of bleached region, whole cell, and background regions were performed using ImageJ. FRAP recovery curves were normalized using EasyFRAP web [71]. The mobile fraction was calculated using the final intensity of normalized recovery curve.

### Confocal live-cell imaging

Live-cell imaging was performed on a Nikon spinning disk confocal CSU-X microscope (Nikon Europe B.V., Düsseldorf, NRW, Germany) equipped with a temperature stage at 37 °C and a 5% CO_2_ saturation. A planar Apo objective 60x oil, NA 1.49 was used. Excitation wavelengths were: 405 nm for BFP; 488 nm for EGFP; 561 nm for mCherry. Image Analysis was done using ImageJ (Version: 1.8.0_172/1.53c).

For N-WASP condensation experiments, HEK293 cells were transfected with 2 µg of pCry2-mCh-N-WASP plasmid using Lipofectamine 2000. After 20 h, cells were imaged by confocal live-cell imaging (561 nm for mCherry) before and after photoactivation for 1 min (488 nm for Cry2, laser intensity at 2.4 mW output).

### Gene ontology biological process pathway enrichment analysis by ClueGO and WormCat

Alpha-synuclein interaction partners were subjected to pathway enrichment analysis by ClueGO (v2.5.9), a plug-in application in Cytoscape [72] (v.3.9.1) (https://cytoscape.org,). The following parameters were used for ClueGO analysis, analysis mode: ClueGO (Functional Analysis); Loaded Marker List (*Homo sapiens* - 9606); ClueGO settings: Ontologies/Pathways (GO GO_BiologicalProcess-EBI-UniProt-GOA-ACAP-ARAP_25.05.2022), Evidence: All; Network specificity: medium; use of GO term Fusion; *p* value threshold ≤ 0.05 for pathway significance; GO Tree interval: 3 (minimum) and 8 (maximum) levels, GO term/pathway selection: 3 genes (minimum), 4% genes, and kappa score of 0.4, for GO term, pathway connectivity.

WormbaseIDs from upregulated and downregulated proteins obtained from proteomic analysis of nematode samples were entered into the online tool WormCat 2.0 [73]. Significant Category 2 pathways were plotted using *R* (v. 4.3.1).

### Lifespan analysis

Synchronized *C. elegans* populations were obtained by incubating gravid adult nematodes with hypochlorite solution, with the resulting eggs that were then transferred onto bacteria-seeded NGM plates. At L4/young adult stage, at least 180 animals (30 animals per plate) were transferred to fresh plates. Animals were transferred every 2 days until egg laying ceased, in order to eliminate offspring. Scoring for dead animals was performed every second day. Death was counted as a lack of touch-evoked movement, and censored animals were counted due to abnormal death (e.g., due to internal hatching). Survival curves were plotted and statistical analysis was performed using GraphPad Prism (GraphPad Software Inc., San Diego, USA).

### Human protein-protein interaction profiling

Protein-Protein Interaction Profiling Service was performed on ProtoArray™ Human Protein Microarrays v5.1 (ThermoFisher Scientific, USA) as previously described [31]. Briefly, microarrays were incubated with blocking buffer (50 mM Hepes, 200 mM NaCl, 0.08% Triton X 100, 25% glycerol, 20 mM glutathione, 1.0 mM DTT, and 1× synthetic block) at 4 °C for 1 h under gentle shaking. Following blocking, arrays were incubated with either monomeric or fibrillar human α-Syn, at two concentrations (5 and 50 ng/μl) diluted in probe buffer (1× PBS, 0.1% Tween-20, and 1× synthetic block) for 90 min at 4 °C. As a positive control, a microarray was incubated with 50 ng/ml of array control protein (*i.e*., yeast calmodulin kinase 1 with a biotin and V5 tags at the N terminus) diluted in probe buffer, and for a negative control one microarray was incubated with probe buffer alone. Following 5 washes with probe buffer at room temperature, microarrays were incubated with anti-α-Syn antibody in probe buffer for 90 min at 4 °C, followed by secondary antibody for 90 min at 4 °C. Following a final wash with probe buffer, arrays were washed with distilled water, dried by centrifugation at 1000 rpm for 1 min, and scanned using an Axon 4000B fluorescent microarray scanner (Molecular Devices). Candidate interactors were considered when the following conditions were satisfied: (a) the fluorescent intensity value was at least 20-fold higher than the corresponding negative control; (b) the normalized fluorescent signal was greater than three standard deviations; (c) the signal-to-noise ratio was higher than 0.5; and (d) the replicate spot coefficient of variation was lower than 50%.

### Immunohistochemistry in cells

Cells on glass coverslips were washed once in PBS before fixing with 4% PFA in PBS for 15 min. Following three washes with PBS, cells were permeabilized and blocked with PBS containing 3% (w/v) BSA, 10 mM glycine, and 0.1% saponin (PBS/B) for 30 min. After blocking, coverslips were washed twice with PBS containing 0.1% saponin (PBS/S). Primary antibody incubation (in PBS/B) was performed for 2 h (dilution 1:500), followed by two PBS/S washes and subsequent incubation with fluorescently labeled secondary antibodies (dilution 1:500) in PBS/B for 45 min. After incubation, coverslips were washed three times with PBS/S and mounted on glass microscope slides using Fluoroshield with DAPI (Sigma, F6057) for imaging. For sample mounting samples, Fluoroshield without DAPI was used (Sigma, F6182). For immunohistochemistry of primary neurons, coverslips were washed once gently with PBS before fixing neurons with 4% PFA in PBS containing 4% (w/v) sucrose, 1 mM MgCl_2_, and 0.1 mM CaCl_2_ for 20 min at room temperature. Following three washes with PBS, cells were permeabilized with PBS containing 0.1% (v/v) Triton-X 100 (PBT) for 5 min. After blocking for 30 min in Blocker™ Casein (Thermo Fisher, 37528) supplemented with 0.1% Triton-X 100 (βCasT), coverslips were washed once in PBT. Primary antibody incubation (dilution: 1:500 in βCasT) was performed for 2 h, followed by two PBT washes and subsequent incubation with fluorescently labeled secondary antibodies (dilution: 1:400 in βCasT) for 45 min. After incubation, the coverslips were washed three times with PBT and mounted on glass microscope slides using Fluoroshield with DAPI (Sigma, F6057) for imaging.

### Oil Red O (ORO) neutral lipid staining

Nematodes were washed off NGM plates, washed 3 times with H_2_O, flash-frozen in liquid nitrogen, and stored at −80 °C until required. On the day prior to staining, ORO stock solution (0.5% w/v in isopropanol) was diluted to 60% in distilled H_2_O and left at room temperature overnight. On the day of staining, ORO solution was filtered twice to remove insoluble precipitate. Pellets were thawed on ice and fixed with 4% PFA at 4 °C on a rotary mixer for 20 min. Fixed worms were washed in PBS, and subsequently dehydrated in 60% isopropanol. To each sample, 1 ml of ORO working solution was added and incubated overnight at room temperature on a rotary mixer. Stained animals were washed 3 times in PBS, and resuspended in 50 µl glycerol, before mounting on a glass slide. Images were acquired using Zeiss Epi-Scope1-Apotome. Staining intensity was measured using ImageJ.

### Oxygen consumption rate (OCR) measurements

OCR was measured using a Seahorse XFe24 Analyzer (Agilent), using a modified protocol as previously described [47, 51]. Briefly, synchronized animals were grown for 5 days at either 20 °C or 25 °C and transferred to freeze-killed OP50 plates for 2 h to empty their gut of live bacteria. Hydrated XFe24 sensor cartridges were calibrated, and the assay was run, at either 20 °C or 25 °C, depending on the initial growth conditions of the animals. Each well of a Seahorse XFe24 Cell Culture Microplate was filled with 500 μl M9 buffer, and 30 animals transferred into each well, with a minimum of 3 wells per condition. OCR measurements were taken at basal conditions, and in response to addition of 20 μM FCCP, followed by 20 mM sodium azide (NaN_3_).

### PALM imaging of neurons and Single Particle Tracking (SPT)

The PALM microscope was a Nikon Ti Eclipse (Nikon France S.A.S., Champigny-sur-Marne, France) equipped with a Perfect Focus System (PFS), a motorized stage TI-S-ER, and an 18zimuthal Ilas² TIRF arm (Gataca Systems, Massy, France) coupled to a laser bench containing 405 nm (100 mW), 491 nm (150 mW), 532 nm (1 W), 561 nm (200 mW) and 642 nm (1 W) diodes. Images were recorded using objective Apo TIRF 100x oil NA 1.49 and a FusionBT sCMOS camera (Hamamatsu Photonics, Massy, France). Photo-conversion experiments were done using the Ilas² scanner system and a 405 nm laser diode while Illumination of the converted mEos3.2 fluorescent protein was exited using the 561 nm laser diode. To record protein trajectories streams of 4000 frames with an exposure time of 50 ms were acquired. The 37 °C atmosphere was created with an incubator box and an air heating system (Life Imaging Services, Basel, Switzerland). This system was controlled by MetaMorph software (Molecular Devices, Sunnyvale, USA).

SPT PALM experiments were analyzed using PALMTracer software, a MetaMorph (Molecular Devices, Sunnyvale, USA) add-on developed at the Interdisciplinary Institute of Neuroscience by Corey Butler (IINS -UMR5297 -CNRS/University of Bordeaux), Adel Mohamed Kechkar (Ecole Nationale Supérieure de Biotechnologie, Constantine, Algeria) and Jean-Baptiste Sibarita (IINS -UMR5297 -CNRS/University of Bordeaux). Briefly, single-molecule localization was achieved using wavelet segmentation, and then filtered out based on the quality of a 2D Gaussian fit. SPT analysis was then done based on the detections using reconnection algorithms and for MSD and D calculations on reconnected trajectories [74, 75].

### Plasmids and DNA cloning

The following plasmids were used: α-synuclein-BFP [19], mCherry-Synapsin 1 and mCherry-(SH3) _A-E_ [12], GFP-N-WASP (Addgene plasmid # 47406; kindly provided by Peter McPherson). mEOS3.2-N-WASP was generated by PCR of N-WASP from GFP-N-WASP (primer fwd: ATCACTCGAGTGAGCTCGGGCCAGCAG, primer rv: ATCAACTGCAGAATTCGAAGCTTTCAG) and subcloning into a pmEOS3.2-C1 backbone using *Xho*I and *Pst*I. pCry2-mCh-N-WASP was generated by restriction enzyme-based cloning (*Kpn*I/*Xho*I) of the N-WASP coding sequence into a pCry2-mCherry backbone plasmid [34].

### Sample preparation, LC–MS/MS measurements, database searching, and SAINT analysis

Co-IP elution fractions were run using SDS-PAGE. In Gel digestion- Molecular weight bands were dissected from 18% SDS PAGE gels of anti-HA CoIP elution fractions derived from Expi293 cells either expressing empty HA-tagged vector or HA-tagged human SNCA wt. Excised gel bands were destained with 1:1 destain solutions A and B (Silverquest kit, #LC6070, Sigma Aldrich), reduced with 10 mM DTT and 2 mM TCEP in 100 mM ammonium bicarbonate and alkylated using 50 mM IAA in 100 mM ammonium bicarbonate for 30 min at room temperature and in the dark. In gel digestion was performed overnight at 30 °C with sufficient Trypsin (20 ng/μl; in 50 mM ammonium bicarbonate) to completely cover the gel pieces. Peptides were extracted from the gel slices by adding an equal volume of extraction buffer (1:2 (vol/vol) of 5% formic acid/ acetronitrile) and incubation at 37 °C with shaking for 15 min. Filter-aided sample preparation- young adult wt(N2), BAN534 *wsp-1(gm324)IV*, BAN550 *wsp-1(gm324)IV;pkIs2386[unc-54p::α-Syn::YFP+unc-119(+)] and* NL5901 *pkIs2386[unc-54p::α-Syn::YFP+unc-119(+)]* animals grown at 20 °C or 25 °C, were collected in water, spun down and stored at -80 °C until further processing. Samples were lysed in 200 μl Lysis buffer (50 mM HEPES (pH 7.4), 150 mM NaCl, 1 mM EDTA, 1.5% SDS, 1 mM DTT; supplemented with 1× protease and phosphatase inhibitor cocktail (ThermoScientific)). Lysis was aided by repeated cycles of sonication in a water bath (6 cycles of 1 min sonication (35 kHz) intermitted by 2 min incubation on ice). Approximately 20 µg of *C. elegans* protein lysates were reduced and alkylated prior to processing by a modified filter-aided sample preparation (FASP) protocol as previously described [76]. Samples were digested overnight with Trypsin (1:20; in 50 mM ammonium bicarbonate) directly on the filters, at 30 °C and precipitated using an equal volume of 2 M KCl for depletion of residual detergents. Tryptic peptides were then cleaned, desalted on C18 stage tips and re-suspended in 20 µl 1% FA for LC-MS analysis. Extracted peptides were dried in a vacuum centrifuge and re-suspended in 30 μl of 5% formic acid for LC-MS analysis. MS runs were performed with at least 3 biological replicates.

Tryptic peptides were analyzed on a Dionex Ultimate 3000 RSLC nanosystem coupled to an Orbitrap Exploris 480 MS. Peptides were injected at starting conditions of 95% eluent A (0.1% FA in water) and 5% eluent B (0.1% FA in 80% ACN), with a flow rate of 300 nL/min. They were loaded onto a trap column cartridge (Acclaim PepMap C18, 100 Å, 5 mm × 300 μm i.d., #160454, Thermo Scientific) and separated by reversed-phase chromatography on an Acclaim PepMap C18, 100 Å, 75 µm × 25 cm (both columns from Thermo Scientific) using a 75 min linear increasing gradient from 5% to 31% of eluent B followed by a 20 min linear increase to 50% eluent B. The mass spectrometer was operated in data dependent and positive ion mode with MS1 spectra recorded at a resolution of 120 K, mass scan range of 375–1550, automatic gain control (AGC) target value of 300% (3 × 10^6^) ions, maxIT of 25 ms, charge state of 2–7, dynamic exclusion of 60 s with exclusion after 1 time and a mass tolerance of 10 ppm. Precursor ions for MS/MS were selected using a top-speed method with a cycle time of 2 s. A decision tree was used to acquire MS2 spectra with a minimum precursor signal intensity threshold of 3 × 10^5^ for scan priority one and an intensity range of 1 × 10^4^ to 3 × 10^5^ for scan priority two. Data-dependent MS2 scan settings were as follows: isolation window of 2 *m*/*z*, normalized collision energy (NCE) of 30% (High-energy Collision Dissociation (HCD), 7.5 K and 15 K resolution, AGC target value of 100% (1 × 10^5^), maxIT set to 20 and 50 ms, for scan priority one and two, respectively. Full MS data were acquired in the profile mode with fragment spectra recorded in the centroid mode.

Raw data files were processed with Proteome Discoverer™ software (v2.5.0.400, Thermo Scientific) using SEQUEST® HT search engine against the Swiss-Prot® *Homo sapiens* (v2021-06-20) or *Caenorhabditis elegans* (v2022-12-14) databases. Peptides were identified by specifying trypsin as the protease, with up to 2 missed cleavage sites allowed and restricting peptide length between 7 and 30 amino acids. Precursor mass tolerance was set to 10 ppm, and fragment mass tolerance to 0.02 Da MS2. Static modifications were set as carbamidomethylated cysteine, while dynamic modifications included methionine and N-terminal loss of methionine, for all searches. Peptide and protein FDR were set to 1% by the peptide and protein validator nodes in the Consensus workflow. Default settings of individual nodes were used if not otherwise specified. In the Spectrum Selector node, the Lowest Charge State = 2 and Highest Charge State = 6 were used. The INFERYS rescoring node was set to automatic mode, and the resulting peptide hits were filtered for maximum 1% FDR using the Percolator algorithm in the Processing workflow. A second-stage search was activated to identify semi-tryptic peptides. Both unique and razor peptides were selected for protein quantification. Proteins identified by site, reverse or potential contaminants were filtered out prior to analysis.

SAINT analysis of SNCA IP was performed as previously described [77]. Briefly PSMs of at least 3 biological replicates of HA only (control) and human SNCA associated protein complexes were uploaded into CRAPome with the following settings: Organism- H. sapiens, Experiment Type- Single step Epitope tag AP MS and Quantitation type- SPC. Probabilistic SAINT Score (SP), SAINT analysis was performed with user controls, averaging of biological replicates and 10 virtual controls. Prey proteins with a Probabilistic SAINT Score (SP) ≥ 0.5 were considered SNCA IP.

Raw MS data are publicly available under the following accession numbers- ProteomeXchange (PXD050719) and jPOST* (JPST002992) [78].

### Sholl analysis

Fixed mDANs on coverslips were washed with PBS, stained with Hoechst, and mounted on glass slides using a fluorescent mounting medium (Dako). Individual cells expressing EGFP (successfully transfected with shRNA) were imaged using LSM900 with a 20x objective. For PFF-treated cells, the presence of Alexa Fluor 647 within the cell was confirmed prior to imaging. Sholl analysis was performed using the ImageJ Neuroanatomy plugin, on maximum Z projections, with a step size of 2um. Graphs were plotted using GraphPad Prism (GraphPad Software Inc., San Diego, USA). A linear mixed effect model was applied for statistical analysis by applying a published code adaption for R [79] and (https://zenodo.org/records/1158612).

### Thrashing assay

Individual animals were transferred into a drop of M9 buffer on a glass slide. Each full-body bends (left to right to left again) were counted for a total of 90 s for each animal. Body bends for 8 animals per strain per experiment were assessed.

### Supplementary information

Supplementary figures and legends

Supplementary table ST1

Supplementary table ST2

Supplementary table ST3

Supplementary table ST4

Supplementary table ST5

Supplementary table ST6

Original data

## Data Availability

Raw MS data are publicly available under the following accession numbers- ProteomeXchange (PXD050719) and jPOST* (JPST002992).
