# Supplementary material for: Actin-nucleation promoting factor N-WASP influences alpha-synuclein condensates and pathology
Source: Cell Death Dis. 2024 Apr 30;15(4):304. doi: 10.1038/s41419-024-06686-7 (PMC11063037; doi:10.1038/s41419-024-06686-7)
Supplement: Supplementary file 1 — Supplementary figures and legends [file 41419_2024_6686_MOESM1_ESM.pdf]

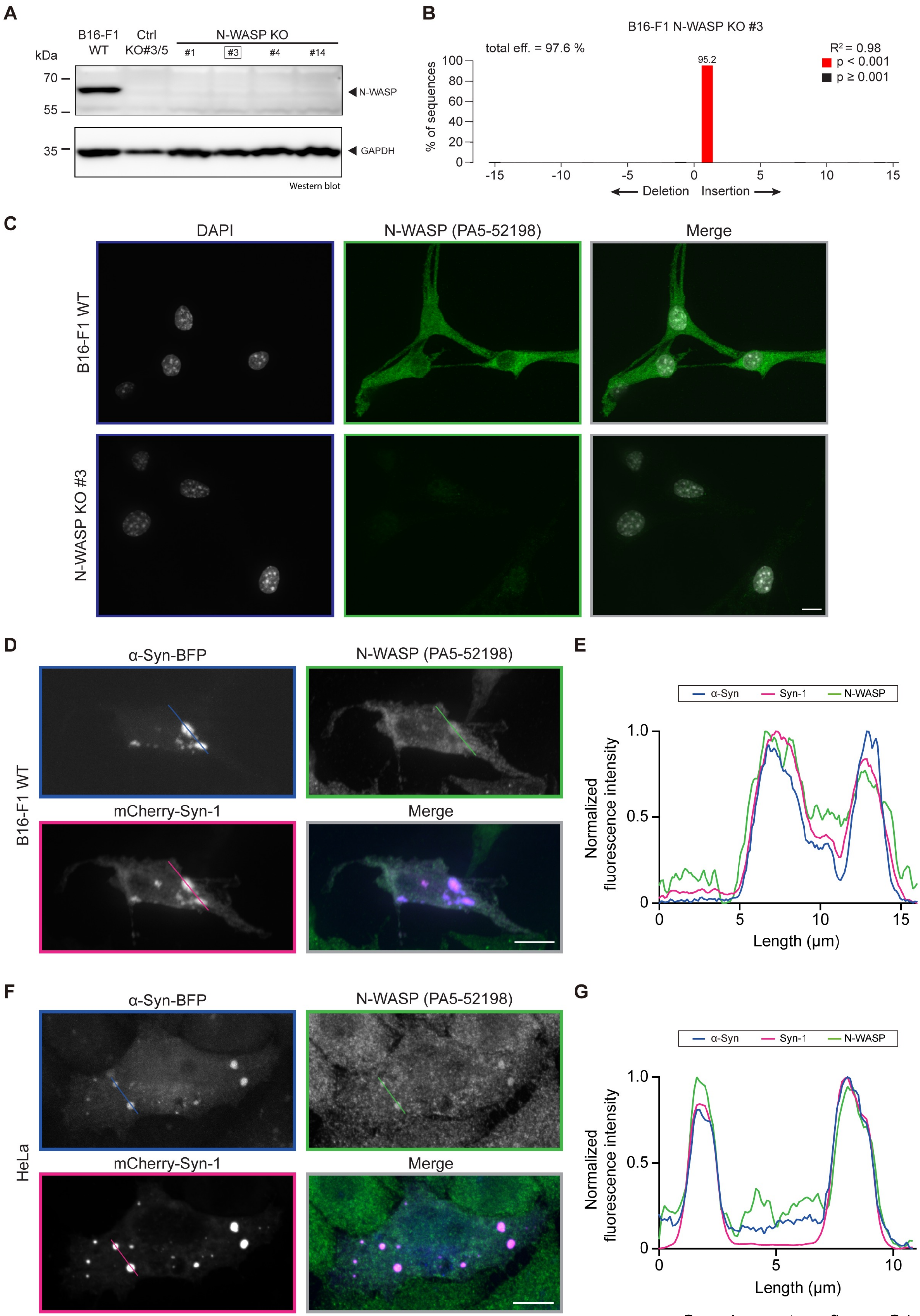


Supplementary figure S1

**Supplementary figure S1 (relative to figure 2).** (A) Western Blot of wild type and N-WASP KO B16-F1 cells. Ctrl KO#3/5 corresponds to a previously characterized triple-KO cell line lacking Sra1/PIR121+N-WASP, and was used as reference. (B) TIDE sequence trace decomposition analysis of the B16-F1 clone selected for experiments (N-WASP KO #3). No wild-type allele was detectable (0), and all alleles present in this clone (N-WASP KO #3) harbored a 1 bp-insertion ($R^2$ indicates fit quality). (C) Representative confocal images of wild-type and N-WASP-KO (clone #3) cells immunostained for N-WASP. Scale bar= 10 µm. (D-G) Immunostaining of N-WASP in (D) wild-type B16-F1 (WT) and (F) Hela cells expressing α-synuclein-BFP and mCherry-Syn1. Panels on the right (E, G) show colocalization analyses of α-Syn-BFP (blue), mCherry-Syn-1 (red) and endogenous N-WASP (green) along the lines indicated in representative confocal images. Scale bars= 10 µm.

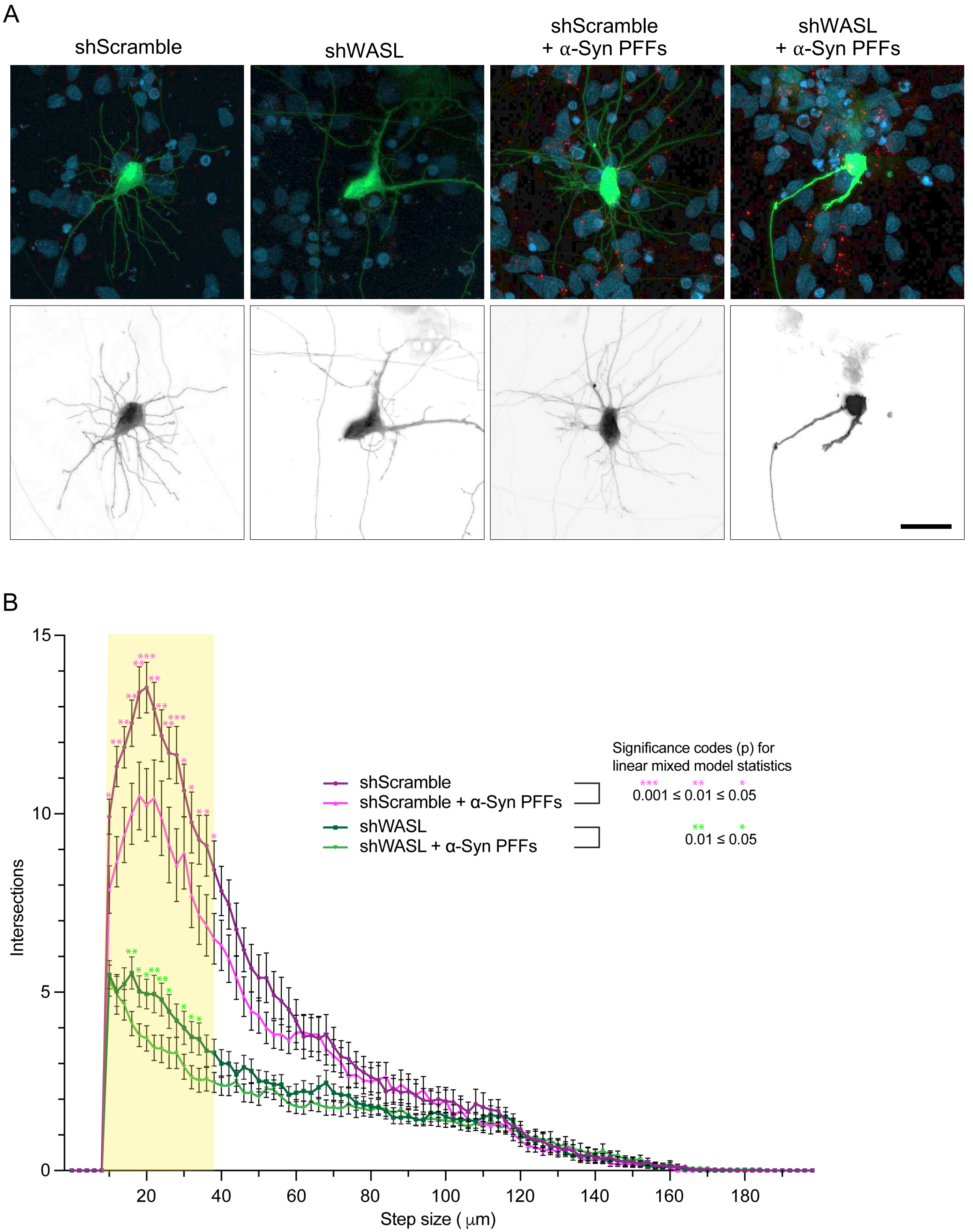


Supplementary figure S2

**Supplementary figure S2 (relative to figure 3).** A) Transfection of shRNA against N-WASP reduces neurite branching in iPSC-derived midbrain dopaminergic neurons (mDANs). α-Syn preformed fibrils (PFFs) have an additive effect to N-WASP downregulation. Top panels show EGFP expression of transfected cells. AF647-labeled PFFs are shown in red, whereas nuclei are in blue (DAPI). Scale bar= 50 µm. (B) Sholl analysis of GFP-positive transfected cells in presence or absence of α-Syn PFFs. Statistics are reported for the four conditions (area of significance shaded in yellow).
