# Supplementary material for: Actin-nucleation promoting factor N-WASP influences alpha-synuclein condensates and pathology
Source: Cell Death Dis. 2024 Apr 30;15(4):304. doi: 10.1038/s41419-024-06686-7 (PMC11063037; doi:10.1038/s41419-024-06686-7)
Supplement: Supplementary file 5 — Supplementary table ST4 [file 41419_2024_6686_MOESM5_ESM.docx]

**Supplementary table ST4.**

**ST4A. Lifespan table with reported individual assays.** Number of animals (dead and censored), median (days) and max (days) lifespan are reported for each individual experiment. To calculate *p* values, Log-rank (Mantel-Cox) test was used.

| **Lifespan at 20 ^o^C** | | | | | |
| --- | --- | --- | --- | --- | --- |
| **Exp** | **Genotypes** | **Dead**  **(Censored)** | **Median**  **(days)** | **Max**  **(days)** | ***p* value** |
| 1 | wt (N2) | 135(45) | 23 | 35 | - |
|  | *wsp-1(gm324)* | 112(128) | 19 | 29 | <0.0001^a^ |
|  | *pkIs2386* | 133(77) | 27 | 35 | <0.0001^a^  <0.0001^b^ |
|  | *wsp-1(gm324);pkIs2386* | 71(169) | 19 | 27 | <0.0001^a^  0.9863^b^  <0.0001^c^ |
| 2 | wt (N2) | 99(61) | 23 | 33 | - |
|  | *wsp-1(gm324)* | 95(115) | 19 | 29 | <0.0001^a^ |
|  | *pkIs2386* | 77(103) | 21 | 35 | 0.8216^a^  0.0003^b^ |
|  | *wsp-1(gm324);pkIs2386* | 55(155) | 17 | 27 | <0.0001^a^  <0.0001^b^  <0.0001^c^ |
| 3 | wt (N2) | 123(57) | 24 | 32 | - |
|  | *wsp-1(gm324)* | 90(120) | 20 | 26 | <0.0001^a^ |
|  | *pkIs2386* | 91(89) | 22 | 36 | 0.2054^a^  <0.0001^b^ |
|  | *wsp-1(gm324);pkIs2386* | 75(135) | 18 | 22 | <0.0001^a^  <0.0001^b^  <0.0001^c^ |
| 4 | wt (N2) | 137(43) | 24 | 32 | - |
|  | *wsp-1(gm324)* | 119(91) | 20 | 26 | <0.0001^a^ |
|  | *pkIs2386* | 91(89) | 26 | 32 | 0.0073^a^  <0.0001^b^ |
|  | *wsp-1(gm324);pkIs2386* | 71(144) | 20 | 24 | <0.0001^a^  <0.0001^b^  <0.0001^c^ |

*^a^p* value compared to wt (N2)

*^b^p* value compared to *wsp-1(gm324)*

*^c^p* value compared to *pkIs2386* overexpressing nematodes

| **Lifespan at 25 ^o^C** | | | | | |
| --- | --- | --- | --- | --- | --- |
| **Exp** | **Genotypes** | **Dead**  **(Censored)** | **Median**  **(days)** | **Max**  **(days)** | ***p* value** |
| 1 | wt (N2) | 110(10) | 16 | 24 | - |
|  | *wsp-1(gm324)* | 161(19) | 13 | 18 | <0.0001^d^ |
|  | *pkIs2386* | 84(66) | 12 | 20 | <0.0001^d^  0.1611^e^ |
|  | *wsp-1(gm324);pkIs2386* | 184(21) | 10 | 15 | <0.0001^d^  <0.0001^e^  <0.0001^f^ |
| 2 | wt (N2) | 72(48) | 14 | 22 | - |
|  | *wsp-1(gm324)* | 180(35) | 12 | 21 | <0.0001^d^ |
|  | *pkIs2386* | 111(69) | 12 | 21 | <0.0001^d^  0.9666^e^ |
|  | *wsp-1(gm324);pkIs2386* | 204(26) | 12 | 16 | <0.0001^d^  <0.0001^e^  <0.0001^f^ |
| 3 | wt (N2) | 157(28) | 16 | 23 | - |
|  | *wsp-1(gm324)* | 145(35) | 13 | 16 | <0.0001^d^ |
|  | *pkIs2386* | 108(72) | 13 | 21 | <0.0001^d^  0.1457^e^ |
|  | *wsp-1(gm324);pkIs2386* | 172(23) | 12 | 16 | <0.0001^d^  <0.0001^e^  <0.0001^f^ |
| 4 | wt (N2) | 136(44) | 16 | 24 | - |
|  | *wsp-1(gm324)* | 110(70) | 12 | 15 | <0.0001^d^ |
|  | *pkIs2386* | 126(54) | 13 | 24 | <0.0001^d^  <0.0001^e^ |
|  | *wsp-1(gm324);pkIs2386* | 82(123) | 10 | 16 | <0.0001^d^  0.0029^e^  <0.0001^f^ |

*^d^p* value compared to wt (N2)

*^e^p* value compared to *wsp-1(gm324)*

*^f^p* value compared to *pkIs2386* overexpressing nematodes

**ST4B. Lifespan assay summary.** Total number of dead and censored animals are calculated by considering all the experiments (N. exp) performed (ST4A). Median±SEM is calculated are reported. Two-way ANOVA (Šidák’s multiple comparisons test, ns=not significant; **p*<0.05, ***p*<0.01) was used to compare the median lifespan of each strain at 20 ^o^C or 25 ^o^C.

| **Lifespan at 20 ^o^C** | | | | | |
| --- | --- | --- | --- | --- | --- |
| **Genotypes** | **Dead**  **(Censored)** | **Median±SEM**  **(days)** | **N. exp** | **Summary** | **Adjusted *p* value** |
| wt (N2) | 494(206) | 23.50±0.29 | 4 | - | - |
| *wsp-1(gm324)* | 416(454) | 19.50±0.29 | 4 | * | 0.0306^a^ |
| *pkIs2386* | 392(358) | 24.00±1.47 | 4 | ns  * | 0.9989^a^  0.0141^b^ |
| *wsp-1(gm324);pkIs2386* | 272(603) | 18.50±0.65 | 4 | **  ns  ** | 0.0066^a^  0.9580^b^  0.0031^c^ |

*^a^p* value compared to wt (N2)

*^b^p* value compared to *wsp-1(gm324)*

*^c^p* value compared to *pkIs2386* overexpressing nematodes

| **Lifespan at 25 ^o^C** | | | | | |
| --- | --- | --- | --- | --- | --- |
| **Genotypes** | **Dead (Censored)** | **Median±SEM**  **(days)** | **N. exp** | **Summary** | **Adjusted *p* value** |
| wt (N2) | 475(130) | 15.50±0.50 | 4 |  | - |
| *wsp-1(gm324)* | 596(159) | 12.50±0.29 | 4 | ** | 0.0018^d^ |
| *pkIs2386* | 429(261) | 12.50±0.29 | 4 | **  ns | 0.0018^d^  >0.9999^e^ |
| *wsp-1(gm324);pkIs2386* | 642(193) | 11.00±0.58 | 4 | ****  ns  ns | <0.0001^d^  0.1200^e^  0.1200^f^ |

*^d^p* value compared to wt (N2)

*^e^p* value compared to *wsp-1(gm324)*

*^f^p* value compared to *pkIs2386* overexpressing nematodes
