# Supplementary figures and images for: Actin-nucleation promoting factor N-WASP influences alpha-synuclein condensates and pathology
Source: Cell Death Dis. 2024 Apr 30;15(4):304. doi: 10.1038/s41419-024-06686-7 (PMC11063037; doi:10.1038/s41419-024-06686-7)

Uncropped blots Fig. 1H-I

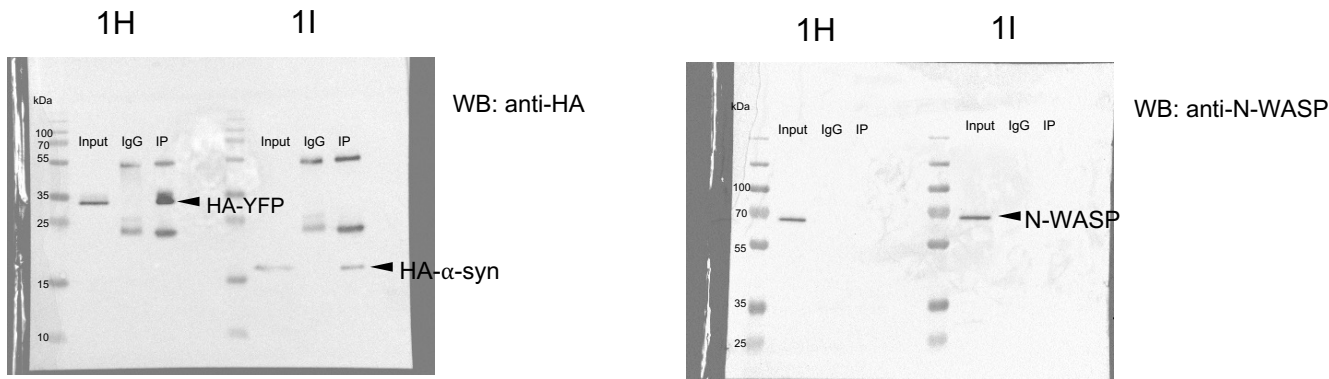

Uncropped blots Fig. 4 F-G

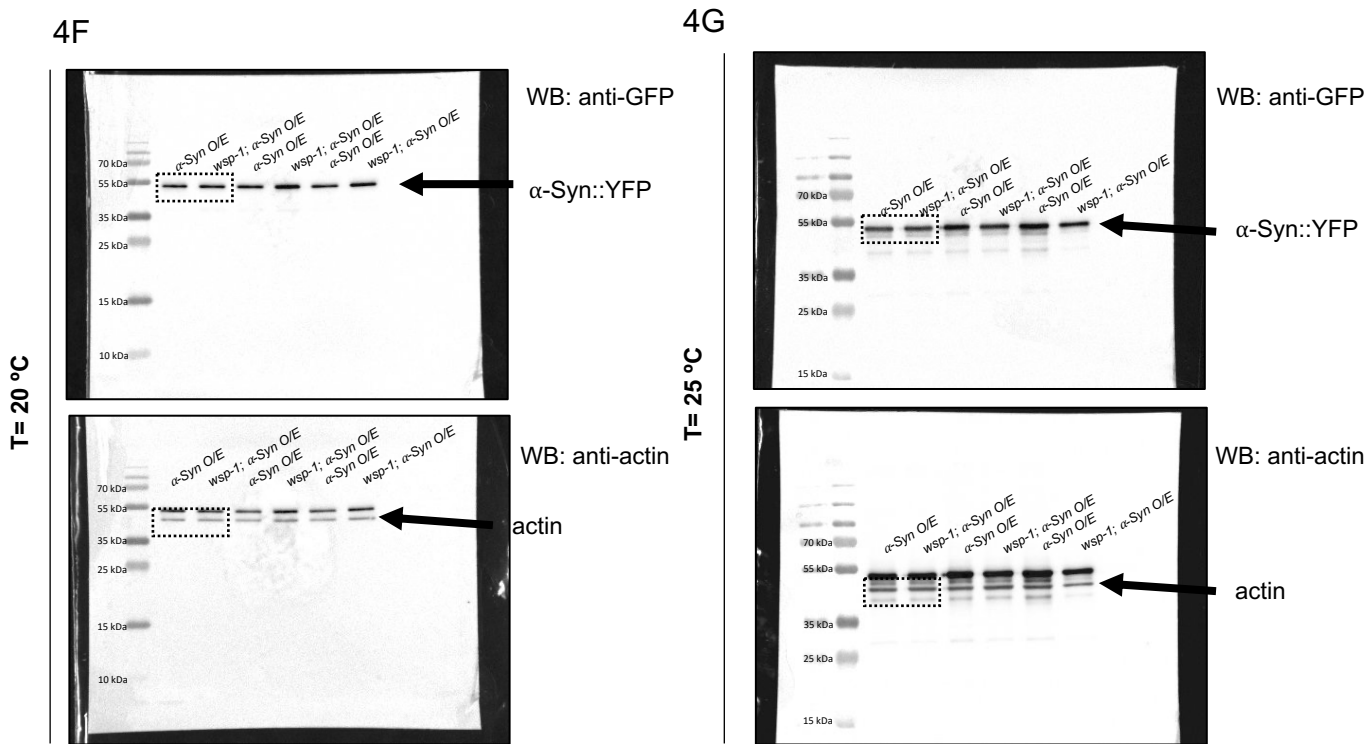

Uncropped blots Fig S1A

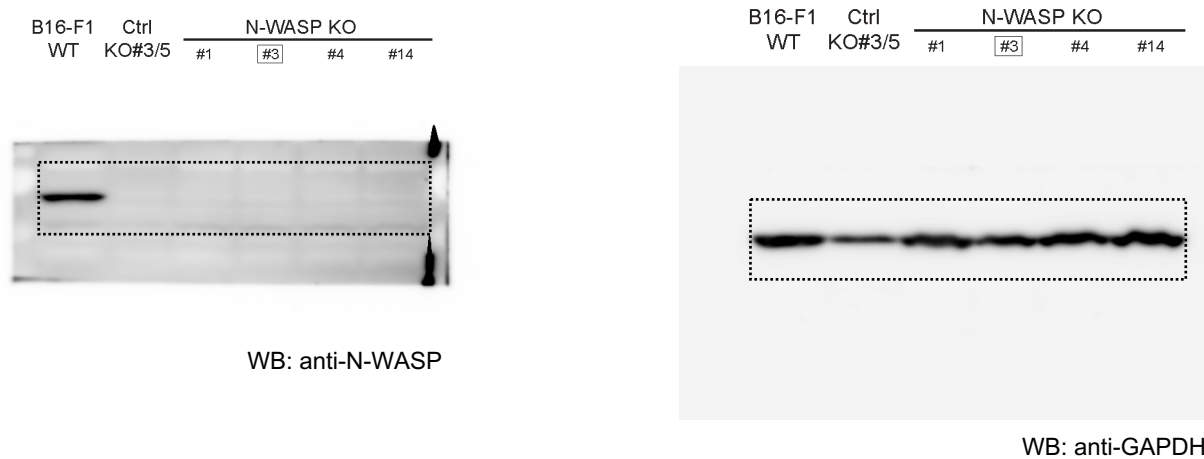

Supplement: Supplementary file 8 — Original data [file 41419_2024_6686_MOESM8_ESM.pdf]
